# Supercritical Fluid Extraction of Plant Flavors and Fragrances

**DOI:** 10.3390/molecules18067194

**Published:** 2013-06-19

**Authors:** Andrea Capuzzo, Massimo E. Maffei, Andrea Occhipinti

**Affiliations:** 1 Department of Life Sciences and Systems Biology, University of Turin, Innovation Centre, Via Quarello 15/A, 10135 Turin, Italy; E-Mails: andrea.capuzzo@unito.it (A.C.); massimo.maffei@unito.it (M.E.M.); 2 Biosfered S.r.l., Academic Spin Off of the University of Turin, Innovation Centre, Via Quarello 15/A, 10135 Turin, Italy

**Keywords:** volatile oils, flavoring compounds, supercritical CO_2_ extraction, modelling, biological activity

## Abstract

Supercritical fluid extraction (SFE) of plant material with solvents like CO_2_, propane, butane, or ethylene is a topic of growing interest. SFE allows the processing of plant material at low temperatures, hence limiting thermal degradation, and avoids the use of toxic solvents. Although today SFE is mainly used for decaffeination of coffee and tea as well as production of hop extracts on a large scale, there is also a growing interest in this extraction method for other industrial applications operating at different scales. In this review we update the literature data on SFE technology, with particular reference to flavors and fragrance, by comparing traditional extraction techniques of some industrial medicinal and aromatic crops with SFE. Moreover, we describe the biological activity of SFE extracts by describing their insecticidal, acaricidal, antimycotic, antimicrobial, cytotoxic and antioxidant properties. Finally, we discuss the process modelling, mass-transfer mechanisms, kinetics parameters and thermodynamic by giving an overview of SFE potential in the flavors and fragrances arena.

## 1. Introduction

### 1.1. Definition and Properties of Supercritical Fluids

Supercritical fluid extraction (SFE) utilizes supercritical fluids, which above their critical point exhibit liquid-like (solvent power, negligible surface tension) as well as gas-like (transport) properties. This property has led in recent years to great interest in supercritical fluids amongst researchers for their various applications. CO_2_ is the supercritical solvent of choice in the extraction of flavor and fragrance compounds, since it is an odorless, colorless, highly pure, safe, cost effective, nontoxic, nonflammable and recyclable gas allowing supercritical operation at relatively low pressures and near room temperature. Generally speaking, supercritical CO_2_ (SC CO_2_) behaves like a lipophilic solvent but, compared to liquid solvents, it has the advantage that its selectivity or solvent power is adjustable and can be set to values ranging from gas-like to liquid-like [1]. The evolution towards Green Analytical Chemistry is to new extraction and sample-preparation processes that should be faster, more reproducible and more environmentally friendly [2]. The techniques involving supercritical fluids are rapid expansion of supercritical fluid extraction (SFE), supercritical solutions (RESS), gas antisolvent process (GAS), supercritical antisolvent process (SAS) and its various modifications, solution enhanced dispersion by supercritical fluids (SEDS), aerosol solvent extraction system (ASES), polymerization-induced phase separation (PIPS), supercritical solvent impregnation (SSI), particles from gas saturated solutions (PGSS) and supercritical assisted atomization (SAA). These processes have been recently reviewed ([3] and references cited therein). The published articles on the usage of SFE mostly for pharmaceutical and food processing accounts for about 45% of the specific scientific literature, the remaining percentage being made by articles dealing with extractions with solvents (about 20%), microwave assisted extraction (MAE, about 10%), ultrasound-assisted extraction (UAE, about 10%) and other extraction techniques which are not yet used for industrial applications in large scale (about 10%).

**Figure 1:**
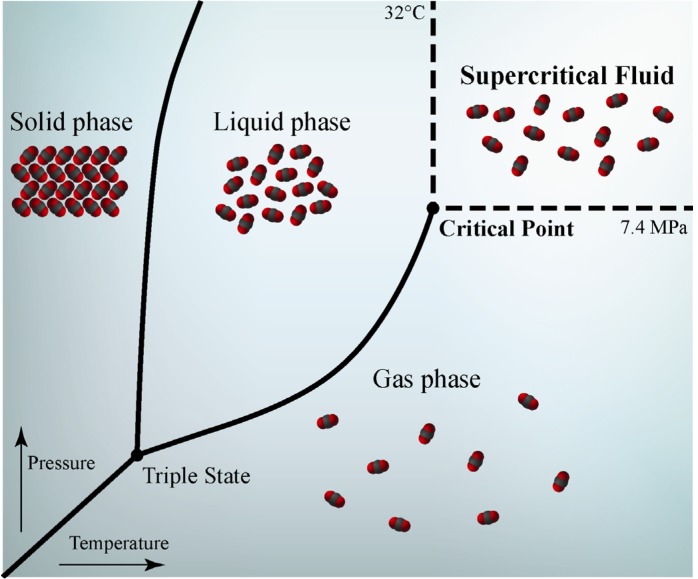
Phase diagram (p, V) for a pure compound in a close system. The triple point indicates the critical pressure and temperature of carbon dioxide.

Thermodynamic relationship of a close system, composed by a single-component, can be described through a phase diagram (p, T) that is the graphic representation of all equilibrium phases for all the p, T coordinates. The diagram region between the triple point, where all the threes physical states (solid, liquid and gas phase) coexist, and the critical point define the p, T coordinates of vapor-liquid equilibrium (Figure 1). Above the critical point (p > pc, T > Tc), the system is a supercritical fluid. Differently from the other part of diagram, the supercritical fluid is not delimited from any transition phase curve, therefore the passage through the critical point is without the exchange of latent heat. Therefore, for a gas at T > Tc but p < pc, every compression will produce an increase of density without formation of liquid and without exchange of latent heat. Over the critical pressure pc, the system passes into the supercritical state 

Supercritical fluids appear as a compressed gas; therefore, supercritical fluids are similar to a liquid with elevated density and low compressibility and at the same time they are similar to a gas with elevated diffusivity and low viscosity. Owing to their high penetration power inside plant materials and their solvent power, supercritical fluids became a good solvent for solutes with chemical compatibility.

### 1.2. Supercritical Fluids for Agronomic and Industrial Applications

The union between economic feasibility and safety are acquiring more consideration and indeed, safer and less harmful solvents that are easy to remove, or recover, are gaining in popularity. The supercritical state of different solvents was investigated (Table 1); however, more than 90% of supercritical fluid extractions have been performed with CO_2_ as supercritical solvent because of the abovementioned practical reasons. SC CO_2_ has a polarity comparable to liquid pentane and, therefore, it is compatible for the solubilization of lipophilic compounds such as lipids and essential oils. However, this low polarity index makes SC CO_2_ hardly suitable for the extraction of polar compounds. To overcome this restriction, practical approaches involve the use of polar co-solvents [2,4,5,6,7]. 

**Table 1:** Critical properties of several solvents used in SFE processes [8].

Solvent	Critical Temperature °C	Critical Pressure MPa
Water	374.0	22.1
Methanol	−34.4	8.0
Carbon dioxide	31.2	7.3
Ethane	32.4	4.8
Nitrous oxide	36.7	7.1
Propane	96.6	4.2

### 1.3. Other Supercritical Fluids

In addition to CO_2_, other supercritical solvents have been evaluated for agronomic applications. There are some reports about the choice of nitrous oxide (N_2_O) as an extraction fluid. The chemical properties of this fluid make N_2_O more suited for the extraction of polar compounds. However, in the presence of a high organic content, the gas can cause violent explosions. This drawback strongly limits its use [9,10,11].

Even water was investigated as a possible supercritical fluid but, unlike CO_2_, the high critical temperature and pressure (Table 1), together with the corrosive nature of H_2_O under these conditions, has limited its practical applications [12]. Even so, water was used in some cases as a co-solvent for the extraction of more polar compounds from aromatic plants. The presence of water as pretreatment of plant material or added to CO_2_ at supercritical and subcritical state as a co-solvent has shown to influence the qualitative and quantitative composition of the extract [13,14,15].

Ethane, propane and dimethyl ether have been used as supercritical solvents for the extraction of bioactive compounds from plants. Beside critical points that are comparable with CO_2_, these solvents have higher polarizability than CO_2_, resulting in a stronger interaction with the more polar compounds and co-solvents [16,17,18]. However, experimental results show that SC CO_2_ offers a wider versatility for the fractionation of extracted compounds using different operative pressures in the extractors or separators [1,7,11,19,20].

### 1.4. Supercritical Fluid Extraction of Flavors and Fragrances

Flavors and fragrances represent a consistent and substantial portion of the world natural product market. Essential oils characterize aromatic plants used in the pharmaceutical, food and fragrance industries. Essential oils contain monoterpene and sesquiterpene hydrocarbons and oxygenated compounds (alcohols, aldehydes, ketones, acids, phenols, oxides, lactones, ethers and esters), which are responsible for the characteristic odors and flavors. The extraction of essential oil components using SFE has received much attention, especially in food, pharmaceutical and cosmetic industries, because it presents an alternative to conventional processes such as organic solvent extraction and steam distillation [10]. SFE of flavors, fragrances and other natural products has been reviewed. These reviews have described a wide range of applications for the extraction of several groups of non-polar compounds, including essential oils, other flavor and fragrance compounds, medicinal compounds, lipids, carotenes and alkaloids, tocopherols and tocotrienols, as well as the global yield and quality of the extracts all of the plant materials investigated, and the possibility of their application in the food, cosmetics and pharmaceutical industries [4,7,11,21,22,23,24,25,26,27,28,29,30]. The nature of extracted plant materials, the mathematical modelling of the process, and the choice of the appropriate model have been reviewed too [31]. 

The use of SFE was demonstrated with a variety of samples including spices, chewing gum, orange peel, spruce needles, and cedar wood [32]. A thoughtful comparison of the extraction kinetic has been established and discussed, in terms of the extraction yields attained in the separators, the variation of the essential oil composition with time and the content of key bioactive substances identified in the different fractions [33,34,35]. Furthermore, to develop and establish novel and effective alternatives to heating treatment, the lethal action of high hydrostatic pressure CO_2_ on microorganisms, with none or only a minimal heating process, has recently received a great deal of attention [36]. 

The aim of this review is to update the state of the art on the application of SFE to flavors and fragrances at both industrial and laboratory scales, by describing mathematical models, biological effects and chemical composition of plant SFE extracts.

## 2. Supercritical Fluid Extraction of Flavors

Conventional extraction with organic solvents has been widely used to obtain natural product extracts, but has drawbacks, such as low selectivity, high energy costs, and the possible loss of volatile compounds during removal of the solvent [37]. Therefore, supercritical fluids are attractive for extracting flavors present in natural materials. Carbon dioxide frequently exhibits an ability to fractionate flavor and aroma components present as complex mixtures of compounds in such materials as pepper, ginger, allspice, and other spices [38]. Below we review some of the most interesting applications of SFE technology for flavors used in industrial processes.

**Figure 2:**
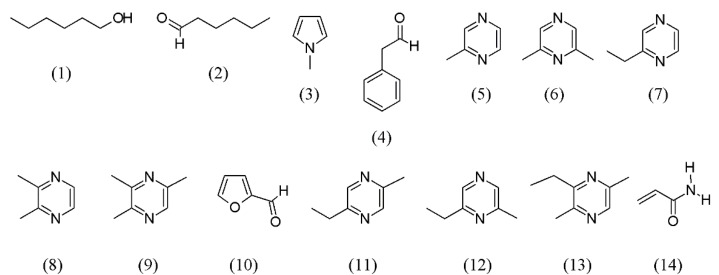
Volatile flavor components of *Arachis hypogaea* roasted peanuts.

A method for SFE extraction and identification of volatile flavor components in roasted peanuts (*Arachis hypogaea*) has been described. Appropriate choice of CO_2_ supercritical fluid density (0.35 g/mL) and extraction temperature (50 °C), at a pressure of 9.6 MPa, provided a selective extraction of compounds associated with roasted flavor rather than nonvolatile lipid material. The compounds were hexanol (**1**), hexanal (**2**), methylpyrrole (**3**), benzene acetaldehyde (**4**), methylpyrazine (**5**), 2,6-dimethylpyrazine (**6**), ethylpyrazine (**7**), 2,3-dimethylpyrazine (**8**), 2,3,5-trimethylpyrazine (**9**), 2-furancarboxaldehyde (**10**), 2-ethyl-5-methyl- (**11**) and 2-ethyl-6-methylpyrazine (**12**), and 3-ethyl-2,5-dimethylpyrazine (**13**) (see Figure 2).

Careful grinding of frozen samples and alternate layering with silanized glass wool in the extraction thimble allowed a good recovery of the volatiles (>85%) in a single extraction step [39]. Considering the importance of tropical almond nuts as a snack item, a study was also conducted to identify the flavor volatiles and acrylamide generated during the roasting of the nuts. The SFE flavor components revealed 74 aroma active compounds made up of 27 hydrocarbons, 12 aldehydes, 11 ketones, seven acids, four esters, three alcohols, five furan derivatives, a pyrazine, and two unknown compounds. While low levels of acrylamide (**14**) were obtained in the roasted nuts (8–86 µg kg^−1^), significant (*p* < 0.05) increases in concentration occurred with increased roasting temperature and time. Carboxylic acids were the most abundant volatiles in the roasted almond nuts and less significant (*p* > 0.05) concentration of acrylamide was generated with mild roasting and shorter roasting period (Figure 2) [40].

*Mucuna* is a genus of around 100 accepted species of climbing vines and shrubs of the family Fabaceae, found worldwide in the woodlands of tropical areas. Three different varieties of *Mucuna* (*aterrima*, *cinerium*, *deeringiana*) and solvents (SC CO_2_, dichloromethane, hexane) were compared. The experiments under supercritical CO_2_ conditions were performed in a laboratory scale unit at 40 and 60 °C over a pressure range from 15 to 25 MPa. A constant flow rate of CO_2_ close to 3 mL min^−1^ was used. The results revealed that temperature, pressure and density were important variables for CO_2_ extraction. The concentration of L-dopa in the defatted meal from SFE was always higher than those without oil extraction or extracted using organic solvents. Linoleic acid (omega-6) was the major oil component and the content of free fatty acids in the oil extracted with supercritical CO_2_ was close to 5% [41]. Recent developments in methods for isolation and measurement of volatiles from cereals have been reviewed; however, SFE has not yet been fully evaluated for cereals [42]. 

Various extraction methods exist for the investigation of aroma components of coffee. Five different coffee extraction methods have been compared, including SFE [43]. Furthermore, the removal of acrylamide from coffee through supercritical CO_2_ extraction has been investigated. The efficiency of acrylamide removal was checked by measuring its content in the coffee beans before and after the supercritical treatment. The supercritical treatment did not affect the caffeine content of coffee and a maximum extraction efficiency of 79% was found for acrylamide. While a pressure variation did not significantly affect the results, temperature affected the extraction process at the highest extent. The addition of ethanol resulted in a significant increase in the extraction performance due to the change in polarity of the supercritical solvent mixture. The best working conditions in the experimental range here investigated were 100 °C, 20 MPa and 9.5% w/w ethanol [44].

Developing low-fat cheese with flavor to match that of full-fat cheese has been a challenge in the dairy industry. Lower fat cheddar and Parmesan grated cheeses have been produced by using SC CO_2_ extraction. Two levels of treatment for each pressure (20 and 35 MPa), temperature (35 and 40 °C) and CO_2_ level (500 and 1,000 g) for each extraction trial were studied. The most efficient parameters for lipid removal resulted in 51.00% fat reduction (wet basis) for cheddar extracted at 20 MPa, 40 °C, 1,000 g CO_2_, and 55.56% fat reduction for Parmesan extracted at 35 MPa, 35 °C, 1,000 g CO_2_. cheddar and Parmesan cheeses showed only nonpolar lipids (triaclyglycerides and free fatty acids) in the recovered lipids extracted by SFE; indicating that polar lipids such as phospholipids are being retained in the cheese matrix [45]. Thus, SFE technology can be used in the dairy industry to develop cheese products lower in fat, which retain flavor compounds that may not be typically fully developed with alternative methods of low-fat cheese processing.

**Figure 3:**
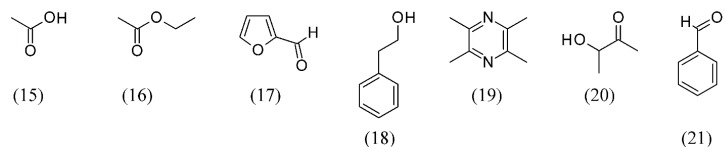
Main aroma compounds found in Zhenjiang aromatic vinegar SFE extracts.

Zhenjiang aromatic vinegar is produced from sticky rice through solid-state fermentation, and is highly prized as one of the four famous China-style vinegars, owing to its unique flavor. SFE was used to recover aroma compounds from Zhenjiang aromatic vinegar. The optimal conditions for the extraction of aroma compounds by SFE were found to be a 25 L h^−1^ CO_2_ flow rate, 35 MPa extraction pressure, and 50 °C extraction temperature. A total of 44 aroma compounds were identified in Zhenjiang aromatic vinegar SFE extract. Acetic acid (**15**), ethyl acetate (**16**), furfural (**17**), phenethyl alcohol (**18**), tetramethylpyrazine (**19**), 3-hydroxy-2-butanone (**20**) and benzaldehyde (**21**) were the main aroma compounds in the vinegar SFE extract (Figure 3) [46].

Cumin is a flowering plant in the family Apiaceae, native from the east Mediterranean to India. Ground cumin is used as a flavoring agent in a number of ethnic cuisines and the quantity of its flavor is commonly the measure of the quality of this spice. For several decades, the spice industry has used a classical distillation procedure for the determination of volatile oil in cumin and other spices. However, the method is cumbersome and requires nearly 8 h to complete. SFE with capillary gas chromatography-flame ionization detection was utilized in the formulation of a rapid, accurate, and specific method for the determination of volatile oil in ground cumin. Samples extracted in a static-dynamic mode with CO_2_ at 55 MPa and 100 °C showed results comparable with those obtained by the official procedure but with much shorter times of extraction [47].

Turmeric (*Curcuma longa*) is a common species whose roots are used in dairy food as colorant and flavoring substitute for saffron. Turmeric contains curcuminoids that have antimutagenic and antioxidant activities, and is thus used for the formulation of foods for the prevention of cancer. Turmeric extracts rich in curcuminoids were obtained by SFE using a mixture of CO_2_ and ethanol, and the assays were performed in a fixed bed extractor at 30 MPa and 30 °C. The supercritical fluid extraction using 50% of the cosolvent that employed the static period method increased the curcuminoid content (0.72% of curcuminoids) and reached a similar extract yield (10%) [48].

SC CO_2_ of onion (*Allium cepa*) flavor was studied using a high pressure apparatus with a 5 L extractor vessel volume. Designed experiments were carried out to map quantitative effects of extraction pressure and temperature on the extraction yield and sulphur recovery. Stagewise precipitations of the extracts carried out using two separators in series provided essential oil rich products with a high-sulphur content [49]. Moreover, ethanol used as a modifier enhanced the yield of onion oil over that obtained by supercritical CO_2_ experiment without a modifier at the CO_2_ flow rate of 1.0 L min^−1^ [50]. The profiles of onion juice extracts revealed the usual thiosulfinates, zwiebelanes, and bissulfine reported in prior studies, as well as cepaenes previously identified in extracts of onion juice through extensive isolation steps and spectroscopic methods [51,52]. SFE of homogenized garlic (*Allium sativum*) shows a good characterization of the major thiosulfinates and small quantities of ajoene, a potent antithrombotic agent. 

**Figure 4:**
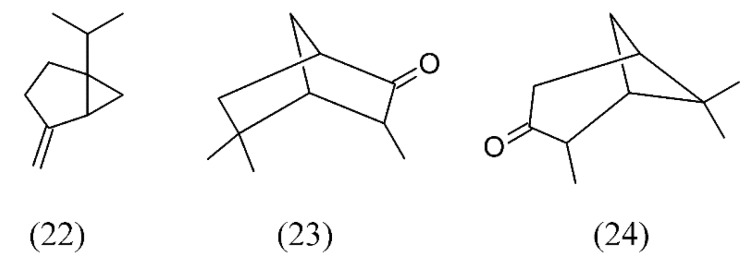
Main components of the hyssop SFE extracts.

*Hyssopus officinalis* (hyssop) is a food ingredient important in flavor industry and in sauce formulations. SFE of hyssop has been performed at various pressures, temperatures, extraction (dynamic and static) times and modifier (methanol) concentrations Main components of the extracts under different SFE conditions were sabinene (**22**), iso-pinocamphone (**23**) and pinocamphone (**24**). The extraction of sabinene (**22**) was favored at 10.13 MPa, 55 °C, 1.5% (v/v) methanol, 30 min dynamic time and 35 min static time. It was found that the use of SFE under different conditions [different temperatures (35, 45, 55, 65 and 75 °C), five different pressure levels (100, 200, 250, 300 and 350 atm), five different static times (15, 20, 25, 30 and 35 min), five different dynamic times (10, 20, 30, 40 and 50 min) and five different modifier amounts (0.0, 1.5, 3.0, 4.5 and 6.0%, v/v)] allowed targeting the extraction of different constituents (Figure 4) [53].

**Figure 5:**
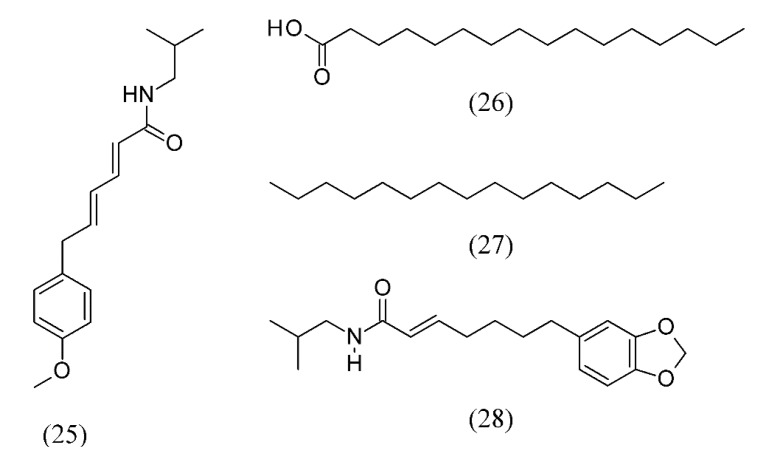
Major components of *Piper piscatorum* SC CO_2_ extracts.

Black pepper (*Piper nigrum*) essential oil has been widely used as a warming and energizing oil that is helpful at the onset of respiratory infections, and for soothing muscular aches and pains. SFE of oil from ground black pepper, using CO_2_ as a solvent, showed a significant increase of extraction rate with increase of pressure or decrease of temperature [54]. The effect of process parameters, namely pressure (7.5, 10, and 15 MPa), temperature (30, 40, and 50 °C) and particle size (0.5 mm, 0.75 mm, and whole berries), on the extraction rate was examined. The essential oil obtained from SC CO_2_ extracts contained higher levels of sequiterpene hydrocarbons, leading to higher sesquiterpene to monoterpene ratios as compared to that obtained from hydrodistillation. The results showed an increase of extraction rate with the increase of pressure or temperature. In contrast, the increase of particle size reduced the extract yield and extraction rate [55]. In this work, the smaller particle size generated a higher yield and grinding was found to liberate more pepper oil by destroying the inner structures of the particles. An increasing yield of pepper mint oil *vs.* size implies that cellular structure should be broken to get a complete extraction of substances. Moreover, even though larger particles contain more essential oil, extraction rate are slower than that of smaller particles, resulting in a longer extraction process. The efficiency of the extraction of fresh and dried leaves of *Piper piscatorum* was evaluated employing SC CO_2_ and co-solvents (10% ethanol and 10% methanol) at 40 °C and 70 °C and a pressure of 40 MPa. The major components of the extracts were piperovatine (**25**), followed by palmitic acid (**26**), pentadecane (**27**) and pipercallosidine (**28**) (Figure 5) [56].

**Figure 6:**
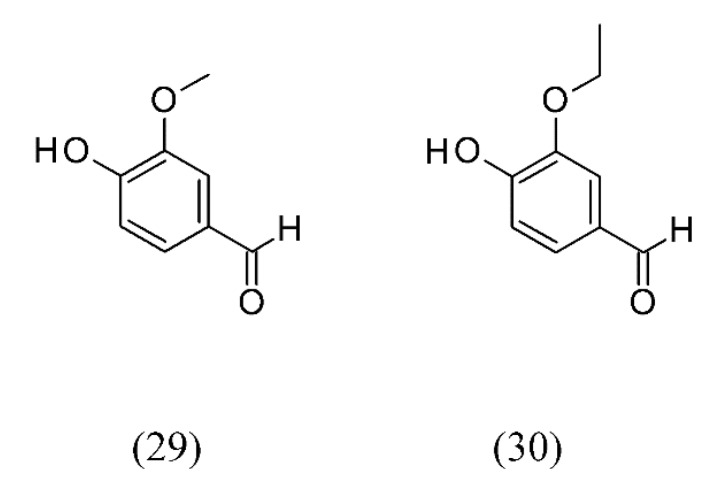
Vanillin and ethyl vanillin from flavored sugars extracted by SC CO_2_.

Extraction of vanillin and ethyl vanillin from flavored sugars with SC CO_2_ (P = 18.9 MPa; T = 45 °C; t = 10 min) under dynamic conditions has been performed. Due to the simple and rapid sample preparation and good average recoveries of 98–104% (concentration range: 10–60 mg) this SFE method was found to be both convenient and reliable for chemical analysis. Since this method does not involve the extraction of sugar, but only of vanillin (**29**) and ethyl vanillin (**30**) (Figure 6), no overloading takes place during liquid chromatography (LC) analyses. Comparing the SFE method to the classical method (Soxhlet) a shorter extraction time (10 minutes by SFE compared to 3–4 hours by Soxhlet) was found and the use of solvent was minimized [57].

Orange oil (from *Citrus sinensis*) is composed largely of terpene hydrocarbons but is also a source of (oxygenated) flavor compounds that are present in low concentrations. To increase the ratio of oxygenated compounds to terpene hydrocarbons, orange oil has been partially fractionated by adsorption of the oxygenated compounds onto porous silica gel, with full utilization of its adsorbent capacity, and then further purified by desorption into SC CO_2_. Extraction at low temperatures and flow rates improved separation. Decanal was concentrated to 20 times that of the feed oil by using at 13.1 MPa, 35 °C, and 2 kg h^−1^ [58].

SFE was optimized for the enrichment and fractionation of the essential oil and the bitter principles of hops (*Humulus lupulus*), both of which contribute to the flavor of beer. The bitter principles, the humulones and lupulones, have been detected and analyzed [59]. Different SFE temperature-pressure combinations were tested for hop essential oil extraction. As a result, the novel hop aroma products were fully compatible with the beer matrix. When added to beer, the novel hop oil preparations imparted a typical, varietal-dependent pleasant hoppy character and increase beer bitterness and mouth feel [60]. In particular, hop SFE was performed in two steps.

Subsequent fractionation of the crude extract from this first SFE step via solid phase extraction (SPE) using octadecylsilica and ethanol/water mixtures, resulted in flavor-active single variety hop oil essence, highly enriched in “floral” compounds of total hop essential oil. When added to a non-aromatized pilot lager, such varietal essences imparted pleasant and pronounced floral, hoppy and citrus scents to the beer flavor. In the second step of the SFE procedure, the hop residue from the first extraction step was further extracted using a CO_2_ density of 0.50 g/mL (11.14 MPa, 50 °C).

As a result of the typical construction of the collection vials and further fractionation of the crude SFE extract via SPE, single variety hop oil essence, highly enriched in oxygenated sesquiterpenoids (“spicy” compounds), were obtained [61]

**Figure 7:**
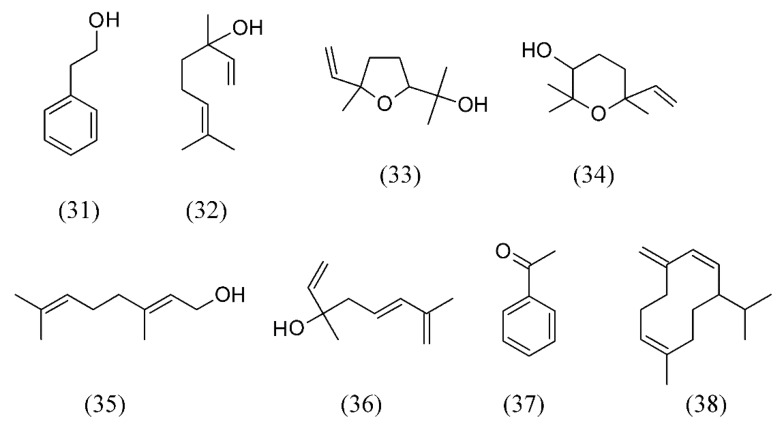
Volatile flavor components of tea flowers (*Camellia sinensis*) isolated by SFE.

Volatile flavor components of tea flowers (*Camellia sinensis*) were isolated by SFE and showed the presence of phenylethanol (**31**), linalool (**32**), (*E*)-linalool oxide furanoid (**33**), epoxy linalool (**34**), geraniol (**35**) and hotrienol (**36**) as the major components. Acetophenone (**37**) and the pheromone germacrene D (**38**) were also. Floral, fresh and fruity odor of tea flowers is retained by SFE with a very little loss of heat sensitive volatiles. The flavor isolated from SFE was found with superior quality compared to distillation (Figure 7) [62]. 

**Figure 8:**
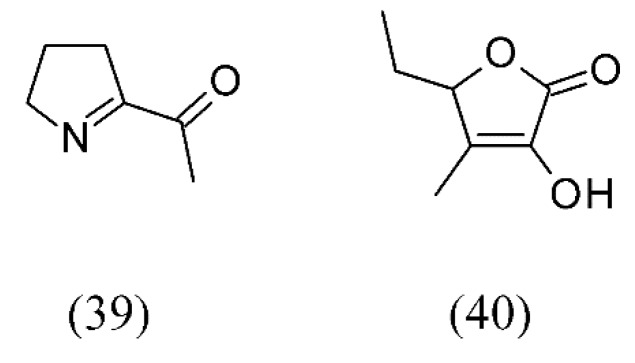
The flavor components of Pandan (*Pandanus amaryllifolius*) leaves extracted by SC CO_2_.

*Pandanus amaryllifolius* is a tropical plant which is commonly used in Southeast Asian cooking as a flavoring. The flavor of pandan leaves was extracted by SC CO_2_ under different conditions of pressure, temperature and contact time to determine the yield of 2-acetyl-1-pyrroline (ACPY, **39**) and various other components; 14 volatile compounds were identified, and the predominant constituents were ACPY and 3-methyl-2(5H)-furanone (**40**) (Figure 8).

The interaction of different conditions significantly influenced the yield of ACPY and various volatile compounds. There is a potential for high yield of ACPY by SC CO_2_ at 20 MPa, 500 °C and 20 min [63].

Ginger (*Zingiber officinale*) produces a hot, fragrant kitchen spice. The volatile compounds responsible for the flavor of Australian-grown ginger have been extracted using SFE. Both fresh and dried ginger samples have been examined and the major effects of the drying process are a reduction in gingerol (**41**) content, an increase in terpene hydrocarbons and the conversion of some monoterpene alcohols to their corresponding acetates [64]. Moreover, fluid densities above 0.8 g mL^−1^ resulted in the co-extraction of significant amounts of triglycerides.

**Figure 9:**
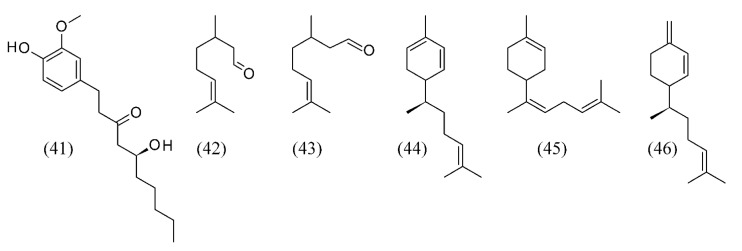
The volatile compounds responsible for the flavor of ginger.

Analysis of the ginger SFE extract by GC-MS indicates that the major components are neral (**42**), geranial (**43**), zingiberene (**44**), α-bisabolene (**45**) and β-sesquiphellandrene (**46**) which together account for 73% of the extract (Figure 9) [65].

SFE was studied as a rapid method for extraction of volatile and semivolatile compounds of plant-derived products such as cigarettes. The method was compared with simultaneous distillation and extraction. SFE was found to extract compounds within a shorter time and avoid the thermal degradation and solvent contamination of samples [66].

A procedure for the recovery of aromatic extracts was also developed for distilled alcoholic beverages by means of a countercurrent SFE. The beverage is directly in contact with the carbon dioxide current in a packed column, and the extracts are recovered in two different fractionation cells, where the depressurization occurs. This method allows the selective extraction of aromatic components of the liquor flavor, rendering a high-value concentrated extract and a colored residue without liquor aroma. Furthermore, the content in ethanol of the aromatic extract can be modified by tuning the extraction/fractionation conditions, rendering from 15 to 95% recovery [67,68].

**Figure 10:**
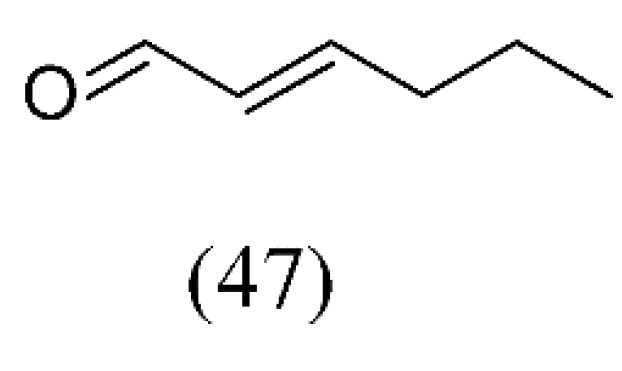
Greenish flavor compound of decaffeinated green tea.

Volatile components in regular and decaffeinated green teas were isolated by simultaneous steam distillation and solvent extraction (SDE). Through a decaffeination process using SC CO_2_ extraction, most volatile components decreased. The more caffeine was removed, the more volatile components were reduced in green teas. In particular, relatively nonpolar components such as terpene-type compounds gradually decreased according to the decaffeination process. Most greenish and floral flavor compounds such as hexanal (**2**), (*E*)-2-hexenal (**47**, Figure 10), and some unknown compounds disappeared or decreased after the decaffeination process [69].

Extraction of oil from cold-press rapeseed cake was performed using SC CO_2_ extraction. The effects of pressure (20, 30, and 40 MPa), temperature (40, 50, and 60 °C), and extraction time (60, 90, and 120 min) on oil yield and composition (tocopherols and carotenoids) were studied using response surface design.

The results indicated that pressure influenced the most the yield of oil, followed by temperature and extraction time. Extraction with SC CO_2_ at 40 MPa and 60 °C was found to be ideal to obtain rapeseed-oil enriched with tocopherols and carotenoids as important functional components [70].

Whey protein isolate contains >90% protein but has flavors that are disliked by some consumers. SFE extractions with a higher temperature and a higher pressure for a longer time were more effective in removing volatiles, and most peaks on the chromatogram of unprocessed WPI sample disappeared or were reduced significantly after SC CO_2_ at all studied conditions. Therefore, SC CO_2_ extraction may provide a green approach to improve flavor quality of whey protein ingredients for novel food applications [71].

## 3. Supercritical Fluid Extraction of Fragrances

Although classical sample preparation procedures are mostly used in labs, the new trends in sample preparation that provide more effective analyte extractions from these complex matrices are gradually being introduced [72]. SFE has been widely applied for the extraction of fragrances in essential oil-producing plants. Some examples will be given for the most representative plant species.

**Figure 11:**
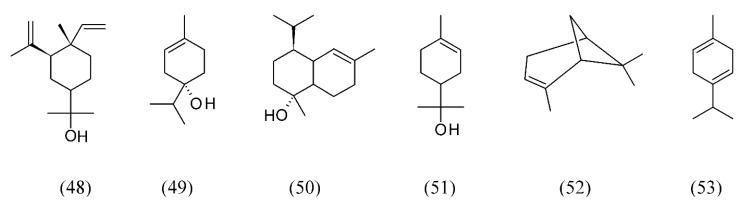
Volatiles from *Abies koreana* needle leaves were extracted by SFE.

*Abies koreana* is a shrub or broadly pyramidal evergreen tree endemic in the mountainous regions of South Korea. Volatiles from alpine needle leaves were extracted by SFE. The major components extracted were elemol (**48**), terpinen-4-ol (**49**), sabinene (**22**, Figure 4), 10(15)-cadinen-4-ol (**50**), α-terpineol (**51**), α-pinene (**52**) and γ-terpinene (**53**) (Figure 11) [73].

**Figure 12:**
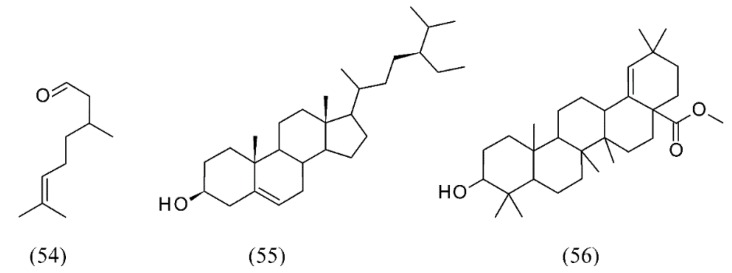
Volatiles from *Eucalyptus* SFE extracts.

*Eucalyptus globulus* is an evergreen tree, one of the most widely cultivated trees native to Australia. SC CO_2_ extraction was carried out at different temperatures, pressures, and ethanol contents to study triterpenic acids of *E. globulus* deciduous bark. The best conditions were 20 MPa, 40°C and 5% ethanol, providing 1.2% (wt.) extraction yield and a 50% concentration of triterpenic acids (5.1 g/kg of bark) [74]. Volatiles were also extracted by SFE from *E. citriodora* leaves. Citronellal (**54**), the major component, was highly extracted (79%). Although the SFE produced lower yields than hydrodistillation, the authors found that its extract was superior in quality in terms of higher concentration of citronellal [75]. Inner and outer barks of *E. grandis* x *globulus* were also extracted by SFE. The two bark fractions showed different chemical compositions. β-Sitosterol (**55**) was the most abundant compound in the inner bark, while long chain aliphatic alcohols were the main family. In the outer bark fraction, triterpenic compounds were the most abundant ones from which methyl morolate (methyl 3-hydroxyolean-18-en-28-oate) (**56**) (Figure 12), identified for the first time as a component of Eucalyptus bark, was the chief component. SFE of methyl morolate with supercritical CO_2_ was obtained at 20 MPa and 60 °C for 6h [76].

**Figure 13:**
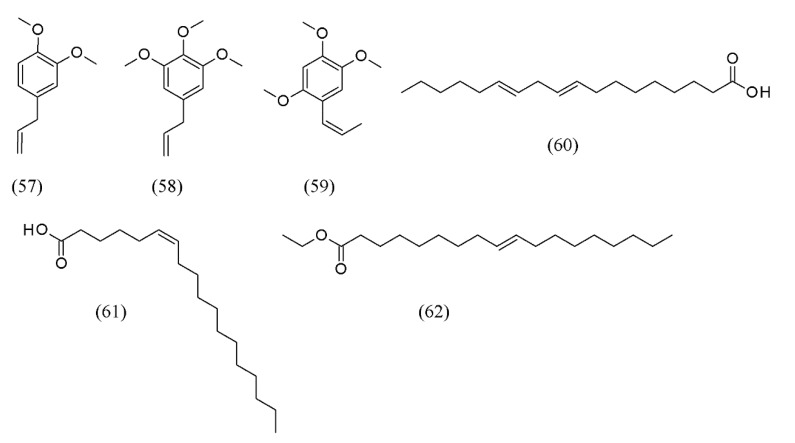
Volatile oils extracted by SFE from *Polygala senega* and *Acorus tatarinowii*.

Volatile oils were extracted from *Polygala senega* and *Acorus tatarinowii*, and a mixture of the two herbs, by using SC CO_2_ extraction. The optimized SFE conditions were 45 MPa at 35 °C for 2 h. Twenty-four compounds were identified in the extract from *P. senega* and *A. tatarinowii* mixture, and six of these had relative contents >1%. These compounds were methyl eugenol (**57**), 1,2,3-trimethoxy-5-(2-propenyl)-benzene (**58**), β-asarone (**59**), (*Z,Z*)-9,12-octadecadienoic acid (**60**), (*Z*)-6-octadecenoic acid (**61**), and ethyl oleate (**62**) (Figure 13). It was interesting to note that with SFE, the combination of the herbs increased the number of pharmacologically active substances in the extract and decreased the number of compounds with one benzene ring compared with the extracts from the individual herbs [77].

**Figure 14:**
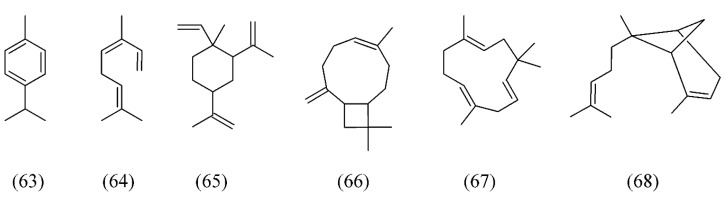
Volatiles of *Bidens tripartita* SC CO_2_ extracts.

*Bidens tripartita*, commonly known as three-lobe beggarticks, is native to large parts of the Northern hemisphere, including Europe, the Indian subcontinent, North America, temperate east Asia, and slightly into northern Africa. The variation of essential oils composition of *B. tripartita* was studied after SC CO_2_ extraction. Volatiles of *B. tripartita* were characterized by the presence of α-pinene (**52**, Figure 11), *p*-cymene (**63**), (*E*)-β-ocimene (**64**), β-elemene (**65**), *iso*-caryophyllene (**66**), α-caryophyllene (**67**), and α-bergamotene (**68**) (Figure 14) [78].

The volatile oil parts of frankincense (*Boswellia carterii*) was extracted with SC CO_2_ under constant pressure (15, 20, or 25 MPa) and fixed temperature (40, 50, or 60 °C) at given times (60, 90, or 120 min) aiming at the acquisition of enriched fractions containing octyl acetate, a compound of pharmaceutical interest. This study demonstrates that SFE is a feasible method for selective acquisition of volatile oil from *B. carterii* being 20 Mpa, 55 °C and 94 min the best conditions to obtain the target compounds in higher amounts [79].

The optimization of SFE of volatile oils and cannabinoids from marihuana (*Cannabis sativa* var. *indica*) has been accomplished. SFE allowed a deterpenation of the plant and a subsequent cannabinoid extraction. For this purpose, pressure, temperature, flow and co-solvent percentage were optimized and the optimal working conditions were at 10 MPa, 35 °C, 1 mL min^−1^, with no co-solvent for the terpenes and 20% of ethanol for the cannabinoids [80].

**Figure 15:**
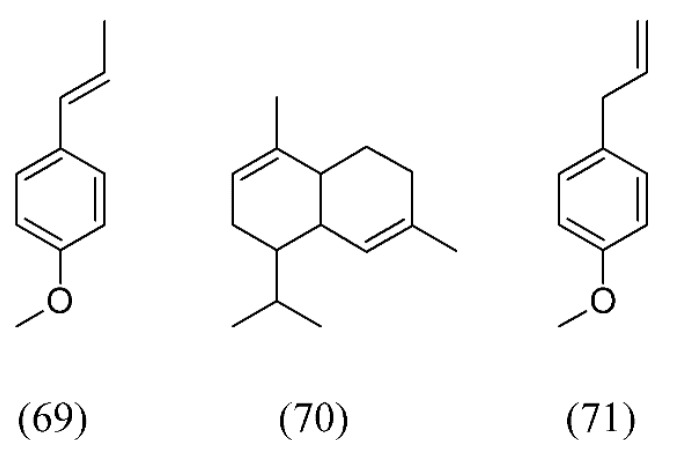
SFE volatile oil components of *Croton zehntneri*.

*Croton zehntneri* is indigenous to the Northeastern of Brazil and particularly rich in the monoterpene (*E*)-anethole (**69**). SFE maximum solubility was observed at 15 °C and 6.67 MPa while the maximum global yield was detected at 20°C at the same pressure. The SFE volatile oil was formed predominantly by (*E)*-anethole (**69**), α-muurolene (**70**), methyl chavicol or estragole (**71**) and germacrene D (**38**, Figure 7) (Figure 15) [81].

**Figure 16:**
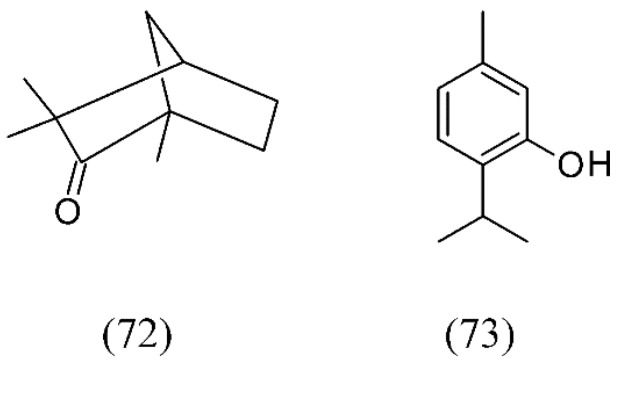
Volatile oil SFE extracts from fennel seeds and thyme leaves.

Volatile oil extracts from fennel seeds (*Foeniculum vulgare*) and thyme leaves (*Thymus vulgaris*) were obtained by SFE. Fennel oil showed the presence of (*E*)-anethole (**69**), estragole (**71**), and fenchone (**72**) as the main components. In contrast, thymol (**73**) and p-cymene (**63**, Figure 14), the most abundant compounds in thyme leaves, were found in SFE extracts as key compounds contributing to the aroma of thyme leaves (Figure 16) [4].

**Figure 17:**
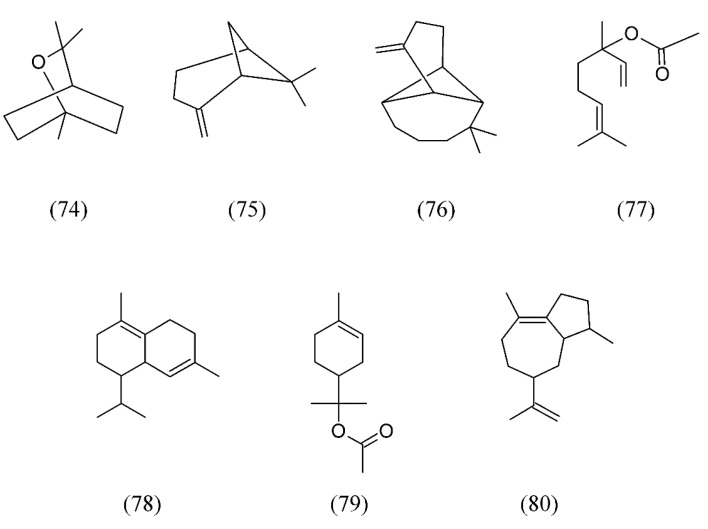
Volatiles from dried berries of *Laurus nobilis* obtained by SC CO_2_ extraction.

The bay laurel (*Laurus nobilis*) is an aromatic evergreen tree or large shrub with green, glossy leaves, native to the Mediterranean region used for bay leaf seasoning in cooking. Isolation of volatile and fixed oils from dried berries of *L. nobilis* were obtained by SC CO_2_ extraction. Experiments carried out at an extraction temperature of 40 °C and pressures of 9 and 25 MPa produced a volatile fraction mainly composed of (*E*)-β-ocimene (**64**, Figure 14), 1,8-cineole (**74**), α-pinene (**52**, Figure 11), β-pinene (**75**), β-longipinene (**76**), linalyl acetate (**77**), δ-cadinene (**78**), α-terpinyl acetate (**79**) and α-bulnesene (**80**) (Figure 17). The last extraction step at 25MPa produced an odorless liquid fraction, in which a very small percentage of fragrance compounds was found, whereas triacylglycerols were dominant [82].

Lavender (*Lavandula* spp) is cultivated extensively in temperate climates as ornamental plant for garden and landscape use, for use as culinary herbs, and commercially for the extraction of essential oils. The effects of three operating conditions of SC CO_2_ extraction, namely pressure, temperature and time, on yield, chemical composition were investigated on *Lavandula angustifolia* using a response surface method coupled with a central composite design. Pressure and time had a significant linear effect on extracts yield, while temperature had a lesser impact except for the effect of its interaction with pressure on extract yield. Generally, the yield of the extracts increased with pressure and time. However, the three operative parameters did not have any impacts on the chemical composition of the extracts [83]. In another work, using absolute calibration, a true quantification of 1,8-cineole (**74**), camphor (**81**), linalool (**32**, Figure 7), linalyl acetate (**77**) and β-caryophyllene (**82**) was carried out after *L. angustifolia* SFE [84]. Finally, volatile oil extracted from *L. angustifolia* using SC CO_2_ by means of a newly developed periodic static-dynamic procedure demonstrated that SFE is a viable technique for separation of constituents such as linalool (**32**, Figure 7), linalyl acetate (**77**, Figure 17), fenchone (**72**), and camphor (**81**) for pharmaceutical and medicinal applications. Furthermore, a substantial reduction of energy consumption and solvent consumption was achieved with this procedure compared to the conventional methods such as such as microwave-accelerated steam distillation and steam distillation for the extraction of essential oil from Italian lavender [85,86].

**Figure 18:**
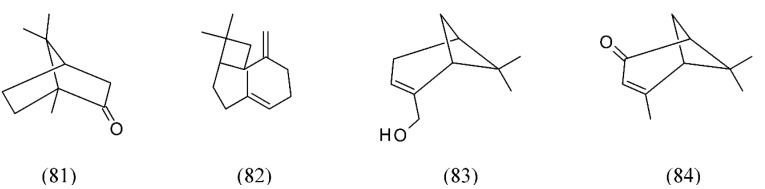
Volatiles from lavender extracted by SC CO_2_.

Lavandin (*L. hybrida*) flowers were extracted using SFE. In this species, to achieve 100% extraction yield, the temperature, pressure, extraction time, and the solvent flow rate were adjusted at 90.6 °C, 6.3 MPa, 30.4 min, and 0.2 mL min^−1^, respectively. The results showed that pressurized fluid extraction is a practical technique for separation of constituents such as 1,8-cineole (**74**), linalool (**32**, Figure 7), linalyl acetate (**77**), and camphor (**81**) from lavandin to be applied in the food, fragrance, pharmaceutical, and natural biocides industries [87]. Finally, chemical profiles of bioactive essential oil and extracts were obtained by SFE, respectively from *L. viridis*. The SFE was performed at 40°C and at extraction pressures of 12 or 18 MPa in two different separators, achieving high yields. Camphor was the main component identified in extracts from the first (1.61 +/− 0.34%) and second SFE separators at 12 MPa. In contrast, the first separator SFE extract at 18 MPa (heavy compounds) was dominated by camphor (**81**) and myrtenol (**83**), whereas the second separator SFE extract (volatiles) was dominated by verbenone (**84**) (Figure 18) [88].

Spearmint (*Mentha spicata*) is a European aromatic plant with an essential oil used in the food, pharmaceutical and cosmetic industries. Since SFE is important for natural products, because it is residue free and preserves thermolabile compounds and product characteristics, it was used to obtain mint volatile oils by sub-/supercritical extraction, with and without modifier and in different operational conditions. The results indicated SFE high yield obtained at 50 °C and 30 MPa, with the crossover of yield isotherms occurring between 14 and 17 MPa.

**Figure 19:**
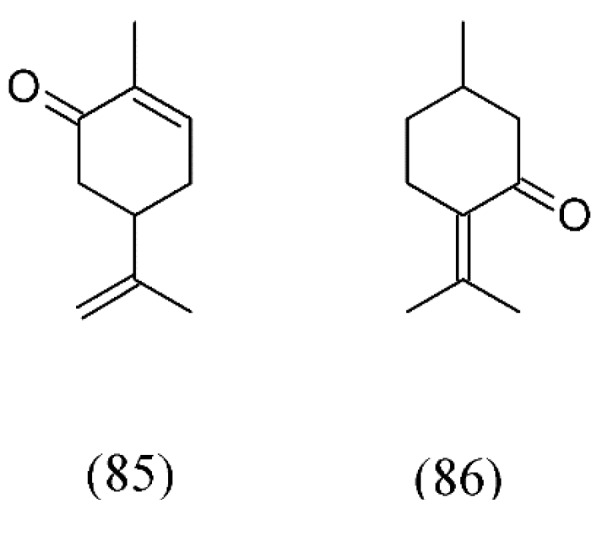
Mint volatiles found in SFE extracts.

When using a co-solvent for SFE, ethanol showed the highest yield compared to ethyl acetate. The mint essential oil was rich in compounds with therapeutic activities and several substances of industrial interest, such as carvone (**85**), 1,8-cineole (**74**, Figure 17), and pulegone (**86**) (Figure 19) [89]. In another work, in order to achieve maximum total yield extraction and SC CO_2_ concentration, tests were done in a laboratorial pilot. The following conditions were considered: pressure 9, 10, 14, and 17 MPa, temperature of 35, 40, 45, 50 °C, mean particles size of 250, 500, 710, and 1000 µm, flow rate at 1, 3, 5, and 8 mL s^−1^ and dynamic time set to 30, 50, 90, and 120 min. The SC CO_2_ extraction optimizing conditions were found to be: 9 MPa, 45 °C, 500 µm, 5 mL s^−1^, 120 min and 9 MPa , 35°C, 250 µm, 1 mL s^−1^, and 30 min [90].

*Ocimum basilicum* (sweet basil) is an important essential oil plant used for different purposes (from food flavoring to pharmaceutical applications) characterized by the presence of several chemotypes. In a comparative analysis between hydrodistillation of essential oils (EO) and SC CO_2_ extracts, SFE was found to yield a higher (4-fold) percentage of 1,8-cineole (**74**), linalool (**32**) (5.8-fold), eugenol (**87**) (1.2-fold) and germacrene D (**38**, Figure 7) (28-fold) with respect to EO. On the other hand, EO composition was characterized by higher percentages of T-cadinol (**88**) (3-fold) and some other sesquiterpenes with respect to SFE [91]. With an extraction vessel of 350 mL and two separators of 250 mL, SFE was also used to carry out studies at temperatures of 40 and 50 °C and pressures of 10 and 12 MPa. The sweet basil oil was analyzed by GC-MS and-its composition was compared with that of the oil isolated by hydrodistillation [92]. Finally, SFE extracts from sweet basil with CO_2_ and the co-solvent H_2_O were performed at 1, 10, and 20% (w/w), at pressures of 10 to 30 MPa at 30 and 40 °C. At 1% of co-solvent, the largest global yield was obtained at 10 MPa and 30 °C; at 10% of co-solvent at 10 and 15 MPa, and at 20% of co-solvent at 30 MPa and 30 °C. The main components identified in the extracts were eugenol (**87**), germacrene D (**38**, Figure 7) and T-cadinol (**88**).

**Figure 20:**
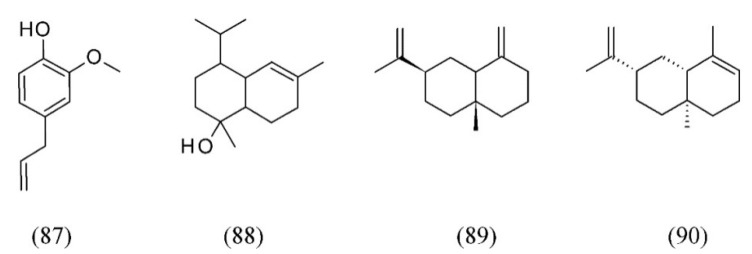
Basil volatiles extracted by SFE.

Three types of SFE extracts from sweet basil were produced, for which the estimated cost of manufacturing (class 5 type) varied from US$ 48 to US$ 1,050 per kilogram of dry extract [13]. Clove basil (*O. gratissimum*) volatile oil was extracted using SFE. Eugenol and β-selinene were the major compounds. The relative proportion of eugenol (**87**) varied from 35 to 60%, while the content of β-selinene (**89**) remained approximately constant (11.5–14.1%, area). The other substances quantified in the extracts were 1,8-cineole (**74**, Figure 17), β-caryophyllene (**82**) and α-selinene (**90**) [93] (Figure 20).

*Peumus boldus* (boldo) is a characteristic component of the sclerophyllous forest endemic to central Chile. Its leaves, which have a strong, woody and slightly bitter flavor and camphor-like aroma, are used for culinary purposes, primarily in Latin America. The leaves are used in a similar manner to bay leaves and also used as a tisane. SC CO_2_ extraction of volatile oils from boldo leaves subjected to rapid decompression of a CO_2_-impregnated sample, conventional milling, and low-temperature milling were studied. Low-temperature conditioning prior to milling decreased heat-driven losses of volatile compounds during milling, as attested by a higher extract yield for low-temperature than conventionally milled sample. Extract yield was even larger for the rapidly decompressed sample [94].

*Salvia officinalis* (sage) is an odorous small perennial shrub native to the Mediterranean region and it is largely cultivated for culinary and medicinal purposes. SFE extraction of sage was investigated and compared to extraction performed by Soxhlet ethanol-water (70:30) mixture extraction (SE) and hydrodistillation (HD). SFE allowed isolation of wide spectrum of phytochemicals, while other applied methods were limited to either volatiles (HD) or high molecular compounds isolation (SE). The volatile fraction could be isolated at low pressure and low CO_2_ consumption, whereby the pressures between 10 and 15 MPa, followed by increased CO_2_ consumption, were favorable for obtaining the desired selectivity of specific terpenes [95,96]. The combination of ultrasound-assisted extraction followed by re-extraction of obtained sage extract with SC CO_2_ was also performed. This procedure gave two valuable products: the ultrasound extract which was rich in sugars and SFE extract which was rich in terpenoids [97]. The influence of process parameters, such CO_2_ density and extraction time, on the composition of sage extracts was also studied. A balance between CO_2_ solvent power and selectivity was required to optimize sage oil composition. Moreover, to obtain volatile oil, the SFE products were fractionated in two separators operated in series. This procedure was required to eliminate co-extracted products like cuticular waxes. The extraction time proved to be one of the main parameters that determine the composition of the oil extracted. Lower-molecular-weight and less polar compounds were more readily extracted with the other families of compounds exhibiting higher diffusion times [98]. Finally, dry sage leaves were extracted with dense CO_2_ under the following conditions: pressure, 9–12.8 MPa; temperature, 25–50 °C; CO_2_ flow rate, 0.05–0.35 g min^−1^; solvent-to-feed ratio, 16:21. The oil in finely ground particles was easily accessible to the solvent and its extraction was controlled by phase equilibrium. Collection efficiency of a cooled glass U-tube at ambient pressure was low for volatile substances but good for sesqui- and diterpenes [99]. An endemic sage of the Sardinian island is *Salvia desoleana*. SC CO_2_ extraction coupled to a fractional separation technique isolated the plant fragrances. The process was carried out by operating at 9 MPa and 50 °C in the extraction vessel, at 90 MPa and below −5°C in the first separator to selectively precipitate the cuticular waxes, and at a pressure of 1.5–2 MPa and temperatures in the range of 15–21 °C in the second separator to recover the volatile oil [100].

**Figure 21:**
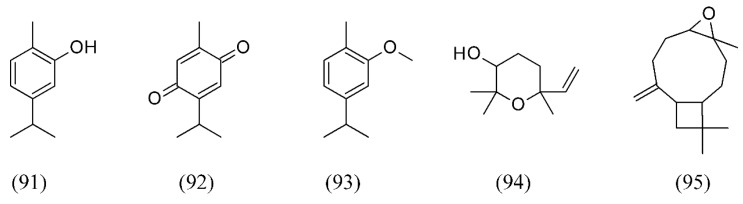
SFE volatile oil compounds from *Thymus* spp.

Thyme (*Thymus vulgaris*) is a common ingredient in cooking and as a herbal medicine. SFE of the volatile oil from *T. vulgaris* aerial flowering parts was performed under different conditions of pressure, temperature, mean particle size and CO_2_ flow rate. The main volatile components obtained were p-cymene (**63**, Figure 14), γ-terpinene (**53**, Figure 11), linalool (**32**, Figure 7), thymol (**73**, Figure 16), and carvacrol (**91**). The main difference was found to be the relative percentage of thymoquinone (**92**) (not found in the essential oil) and carvacrol methyl ether (**93**) [4,101]. SFE at 40 °C and a working pressure of 12 or 18 MPa obtained volatile oil and extracts from the aerial parts of *T. lotocephalus*. Oxygen-containing monoterpenes were the primary constituents in SFE extracts collected in the second separator, while the extracts obtained in the first separator were predominantly oxygen-containing sesquiterpenes. Camphor (**81** Figure 18) and *cis*-linalool oxide (**94**) were the major compounds in the extracts of the second separator obtained at pressures of 12 and 18 MPa, respectively. Caryophyllene oxide (**95**) was the primary constituent identified in the extracts of the first separator (Figure 21) [102].

Table 2 summarizes plant species and major components of flavors and fragrances extracted by SFE.

**Table 2:** Summary of flavors and fragrances extracted by SFE.

Plant species	Raw material	Main compound extracted or process	Co-solvent	Ref.
Flavors				
n/a	cheddar and parmesan cheese	defatted cheese	None	[45]
n/a	cigarettes	volatile and semivolatile compounds	None	[66]
n/a	distilled alcoholic beverages	aromatic components	None	[67]
n/a	flavored sugars	vanillin (29) and ethyl vanillin (30) extraction	None	[57]
n/a	whey protein isolate	volatile removal	None	[71]
*Allium cepa*	onion flavor	essential oil with a high-sulphur content	Ethanol	[49,50]
*Allium sativum*	homogenized garlic	thiosulfinates, zwiebelanes, and bissulfine	None	[51,52]
*Arachis hypogaea*	roasted peanuts	hexanol (1), hexanal (2), methylpyrrole (3), phenyl acetaldehyde (4), methylpyrazine (5), 2,6-dimethylpyrazine (6), ethylpyrazine (7), 2,3-dimethylpyrazine (8), 2,3,5-trimethylpyrazine (9), 2-furancarboxaldehyde (10), 2-ethyl-5-methyl- (11) and 2-ethyl-6-methylpyrazine (12), and 3-ethyl-2,5-dimethyl-pyrazine (13)	None	[39]
*Brassica napus*	cold-press rapeseed cake	tocopherols and carotenoids	None	[70]
*Camellia sinensis*	tea flowers	phenylethanol (31), linalool (32), (*E*)-linalool oxide furanoid (33), epoxy linalool (34), geraniol (35), hotrienol (36), acetophenone (37) and germacrene D (38)	None	[62]
*Camellia sinensis*	decaffeinated green teas	hexanal (2), (*E*)-2-hexenal (47)	None	[69]
*Citrus sinensis*	orange oil	terpene hydrocarbons	None	[58]
*Coffea arabica*	coffee	acrylamide removal	Ethanol	[44]
*Cuminum cyminum*	ground cumin	volatile oil		[47]
*Curcuma longa*	turmeric roots	curcuminoids	Ethanol	[48]
*Humulus lupulus*	hops	humulones and lupulones	None	[59,60,61]
*Hyssopus officinalis*	hyssop	sabinene (22), iso-pinocamphone (23) and pinocamphone (24)	Methanol	[53]
*Mucuna aterrima*, *M. cinerium*, *M. deeringiana*		linoleic acid (omega-6)	None	[41]
*Oryza sativa*	aromatic vinegar from sticky rice	acetic acid (15), ethyl acetate (16), furfural (17), phenethyl alcohol (18), tetramethyl-pyrazine (19), 3-hydroxy-2-butanone (20) and benzaldehyde (21)		[46]
*Pandanus amaryllifolius*	pandan leaves	2-acetyl-1-pyrroline (ACPY) (39) and 3-methyl-2(5H)-furanone (40)	None	[63]
*Piper nigrum*	black pepper	essential oil	None	[54,55]
*Piper piscatorum*	pepper	piperovatine (25), followed by palmitic acid (26), pentadecane (27) and pipercallosidine (28)	Ethanol, methanol	[56]
*Zingiber officinale*	ginger	gingerol (41), neral (42), geranial (43), zingiberene (44), α-bisabolene (45) and β-sesquiphellandrene (46)	None	[64,65]
Fragrances				
*Abies koreana*	needle leaves	elemol (48), terpinen-4-ol (49), sabinene (22), 10(15)-cadinen-4-ol (50), α-terpineol (51), α-pinene (52) and γ-terpinene (53)	None	[73]
*Bidens tripartita*	three-lobe beggarticks	α-pinene (52), *p*-cymene (63), (*E*)-β-ocimene (64), β-elemene (65), *iso*-caryophyllene (66), α-caryophyllene (67), and α-bergamotene (68)	None	[78]
*Boswellia carterii*	frankincense	octyl acetate	None	[73]
*Cannabis sativa var indica*	marihuana	cannabinoids	Ethanol	[80]
*Croton zehntneri*		(*E*)-anethole (69), α-muurolene (70), methyl chavicol or estragole (71) and germacrene D (38)	None	[81]
*Eucalyptus citriodora*	leaves	citronellal (54)	None	[75]
*Eucalyptus globulus*	barks	triterpenic acids	Ethanol	[74]
*Eucalyptus grandis x globulus*	inner and outer barks	β-sitosterol (55), methyl morolate (56)	None	[76]
*Foeniculum vulgare*	fennel seeds	(*E*)-anethole (69), estragole (71), and fenchone (72)	None	[103]
*Laurus nobilis*	bay laurel	(*E*)-β-ocimene (64), 1,8-cineole (74), α-pinene (52), β-pinene (75), β-longipinene (76), linalyl acetate (77), δ-cadinene (78), α-terpinyl acetate (79) and α-bulnesene (80)	None	[82]
*Lavandula angustifolia*	lavender	1,8-cineole (74), camphor (81), linalool (32), linalyl acetate (77), fenchone (72), camphor (81) and β-caryophyllene (82)	None	[83,84,85]
*Lavandula hybrida*	lavandin	1,8-cineole (74), linalool (32), linalyl acetate (77), and camphor (81)	None	[87]
*Lavandula viridis*	lavender	camphor (81), myrtenol (83), verbenone (84)	None	[88]
*Mentha spicata*	spearmint	carvone (85), 1,8-cineole (74), pulegone (86)	None	[89,90]
*Ocimum basilicum*	sweet basil	1,8-cineole (74), linalool (32), eugenol (87), germacrene D (38), T-cadinol (88)	Water	[91,92]
*Ocimum gratissimum*	clove basil	eugenol (87), β-selinene (89), 1,8-cineole (74), β-caryo-phyllene (82), α-selinene (90)	None	[93]
*Peumus boldus*	boldo	volatile oils	None	[94]
*Polygala senega, Acorus Tatarinowii*	mixture of herbs	methyl eugenol (57), 1,2,3-trimethoxy-5-(2-propenyl)-benzene (58), β-asarone (59), (*Z,Z*)-9,12-octadecadienoic acid (60), (*Z*)-6-octadecenoic acid (61), and ethyl oleate (62)	None	[77]
*Salvia desoleana*	sardinian island sage	cuticular waxes and volatile oil	None	[100]
*Salvia officinalis*	sage	mono-, sesqui- and diterpenes	None	[95,96,97,98,99]
*Thymus vulgaris*	thyme leaves	thymol (73) and *p*-cymene (63)	None	[103]
*Thymus vulgaris*	thyme	*p*-cymene (63), γ-terpinene (53), linalool (32), thymol (73), carvacrol (91), thymoquinone (92), carvacrol methyl ether (93), camphor (81), *cis*-linalool oxide (94)	None	[101,102,103]

## 4. Biological Effect of Supercritical Fluid Extracts

### 4.1. Antifungal Activity

Antifungal activities of several medicinal plants have been determined by zone inhibition method by using their essential oils extracted using SC CO_2_ extraction. The strongest *Ganoderma luciderm* inhibition activity was shown by *Mentha arvensis*, *Hibiscus esculentus* and *Acacia concinna* [104]. 

SC CO_2_ extracts of wild *Smyrnium olusatrum* growing in Sardinia (Italy) and in Portugal were isolated from total plant aerial part (umbels containing seeds). The minimal inhibitory concentration (MIC) and the minimal lethal concentration were used to evaluate the antifungal activity against *Candida albicans*, *Candida tropicalis*, *Candida krusei*, *Candida guillermondii*, *Candida parapsilosis*, *Cryptococcus neoformans*, *Trichophyton rubrum*, *Trichophyton mentagrophytes*, *Microsporum canis*, *Microsporum gypseum*, *Epidermophyton floccosum*, *Aspergillus niger*, *Aspergillus fumigatus and Aspergillus flavus*. Extracts were particularly active against dermatophyte strains and *C. neoformans*, with MIC values in the range of 0.32–0.64 mL mL^−1^ [105]. 

SC CO_2_ extracts of *Echinacea angustifolia* were evaluated for their antifungal activity against fungal strain *Botrytis cinerea*, showing EC_50_ and EC_90_ values of 948 and 1,869 µg mL^−1^, 250 µg mL^−1^ MIC, and a minimum fungicidal concentration (MFC) of 2,000 µg mL^−1^ [106].

Port-Orford cedar (*Chamaecyparis lawsoniana*), Alaska yellow cedar (*Chamaecyparis nootkatensis*), and Eastern red cedar (*Juniperus virginiana*) extracted by SC CO_2_ when tested against two common wood decay fungi, brown-rot fungus (*Gloeophyllum trabeum*) and white-rot fungus (*Trametes versicolor*) showed a higher antifungal activity when compared to Soxhlet extraction. Furthermore, *in vitro* studies showed that *C. nootkatensis* extracts had the strongest antifungal activity, followed by *C. lawsoniana*, and *J. virginiana* [107].

SC CO_2_ extracts of *Artemisia argyi* inflorescence show the presence of 1,8-cineole (**74**, Figure 17), caryophyllene oxide (**95**, Figure 21) and camphor (**81**, Figure 18) which exhibit antifungal activity against *Botrytis cinerea* and *Alternaria alternate*, two common storage pathogens of fruits and vegetables. The inhibition of *B. cinerea* and *A. alternate* were 70.8 and 60.5% [108].

**Figure 22:**
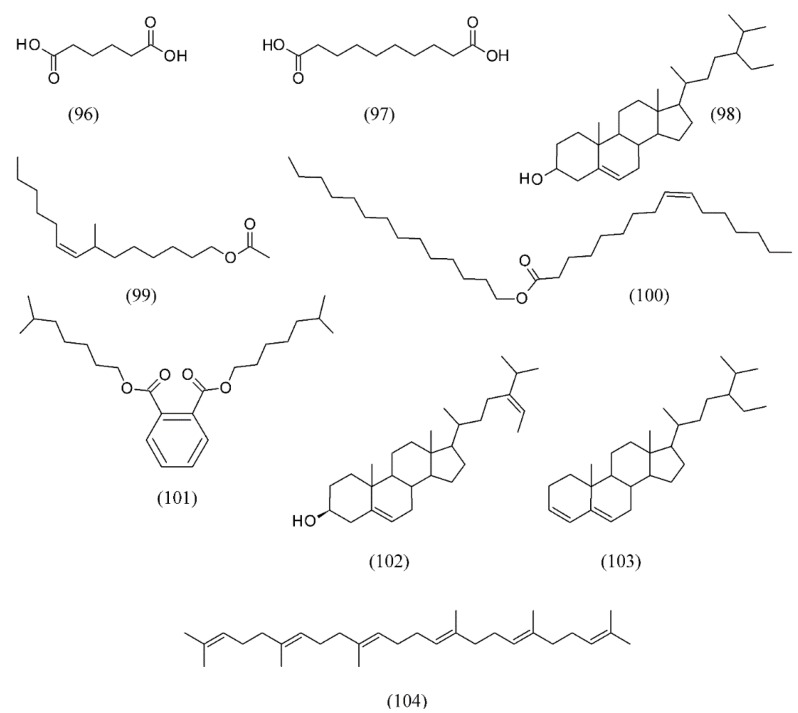
SC CO_2_ extracts of *Stellera chamaejasme* exerting antifungal activity.

The SC CO_2_ extracts of *Stellera chamaejasme* were evaluated by their antifungal activity against *Monilinia fructicola*. The results showed that the SFE extracts exerted a strong antifungal activity against *M. fructicola* with an inhibition ratio of 88.71% at 2,000 μg mL^−1^, minimum inhibitory concentration (MIC) of 250 μg mL^−1^, and minimum fungicidal concentration (MFC) of 2,000 μg mL^−1^. The main active compounds from SFE included hexanedioic acid (**96**), bis (2-ethylhexyl) ester (**97**), sitosterol (**98**), 7-methyl-*Z*-tetradecen-1-ol acetate (**99**), (*Z*)-9-hexadecenoic acid hexadecyl ester (**100**), 1,2-benzenedicarboxylic acid diisooctyl ester (**101**), (3π24Z) stigmasta-5,24(28)-dien-3-ol (**102**), stigmastan-3,5-diene (**103**), and squalene (**104**) (Figure 22) [109].

### 4.2. Insecticidal and Acaricidal Activity

SC CO_2_ extracts from aerial parts of *Tanacetum parthenium* were applied to *Spodoptera littoralis* larvae and showed significant effects on mortality, antifeedancy and growth inhibition. The mortality strongly correlated with feverfew content of terpenoids. SC CO_2_ extracts obtained with pure CO_2_, or with acetone as a co-solvent were more efficient antifeedants and growth inhibitors than the hydrodistilled essential oil alone [110].

Allyl isothiocyanate extracted from *Armoracia rusticana* by SC CO_2_ was found to have insecticidal activity against four major pest species of stored products, maize weevil *Sitophilus zeamais*, lesser grain borer *Rhizopertha dominica*, *Tribolium ferrugineum* and book louse *Liposcelis entomophila*. Allys isothiocyanate obtained from *A. rusticana* was suggested as an alternative to phosphine and methyl bromide against the above four pest species [111].

Extracts from thyme (*Thymus vulgaris*) obtained by SFE, were assessed for their toxicity and antifeedant effects on larvae of Colorado potato beetle, causing mortality in 24 h after their topical application. A strong deterrent effects of extracts against larvae was also observed [112].

The acute toxicity of savory (*Satureja hortensis*) extracts obtained with SC CO_2_ has been tested on larvae of *Musca domestica*, *Spodoptera littoralis*, *Culex quinquefasciatus* and *Leptinotarsa decemlineata* and on adults of *M. domestica*. The efficiency of extract obtained with SC CO_2_ was higher than the efficiency of other extracts, while its extraction yield was by 73% higher than the yield of hydrodistillate [113].

A comparison between traditional extraction techniques (hydrodistillation and organic solvent extraction) and SFE was made for two different populations and crops of *Artemisia absinthium*. The antifeedant activity of SFE extracts was tested on insect pests *Spodoptera littoralis*, *Myzus persicae* and *Rhopalosiphum padi*. SC extracts exhibited stronger antifeedant effects than the traditional ones (up to 8 times more active) [114].

**Figure 23:**
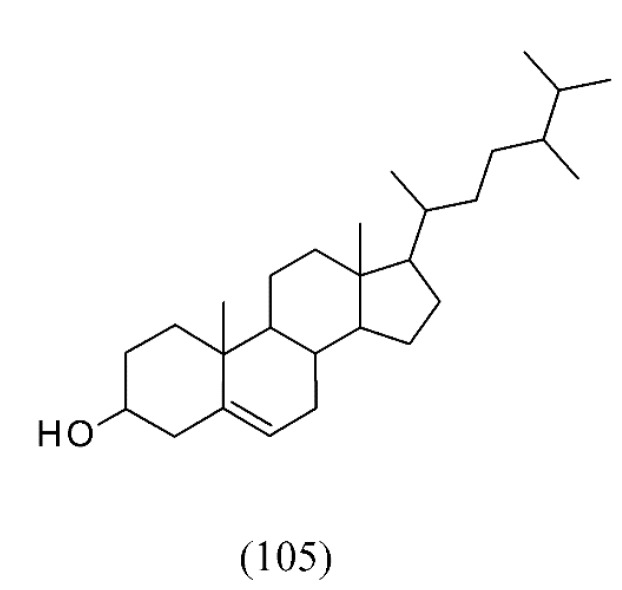
SC CO_2_ extracts of *Stellera chamaejasme* exerting acaricidal activity.

SFE of *Stellera chamaejasme* also showed acaricidal activities against *Tetranychus cinnabarinus*. In this case, the optimal condition of SC CO_2_ extraction was extracting pressure 49 MPa, extracting temperature 15 °C, resolution pressure 43 MPa, and resolution temperature 6.25 °C. SFE extracts of *S. chamaejasme* had contacting and systemic toxicity against *T. cinnabarinus*, which were more active than extracts by cold-soaked extraction method, with LC_50_ value of 2.41 mg mL^−1^ and 3 mg mL^−1^, respectively. Among the major compounds were squalene (**104**, Figure 22) and campesterol (**105**, (Figure 23). Squalene (**104**) showed contacting and systemic toxicity against *T. cinnabarinus*, with LC_50_ value of 9.9 mg mL^−1^ and 12.9 mg mL^−1^, respectively [115].

### 4.3. Antibacterial and Cytotoxic Activity

Emergence of multiresistant strains of bacteria is most commonly connected with the misuse and excessive use of antibiotics. Due to the lack of new antibiotics on the market, studies of antibacterial effect of non-antibiotical substances of different origin, including SFE extracts, are more present nowadays, with the objective to treat humans and animals in cases of infections induced by multiresistant strains of bacteria [116]. 

White grape seeds subjected to sequential SFE showed a strong antibacterial activity against Gram-positive and Gram-negative bacteria *Bacillus cereus*, *Staphylococcus aureus*, *S. coagulansniger*, *Citrobacter freundii*, *Escherichia cloacae*, *E. coli* [117]. 

*Santolina insularis* SC CO_2_ extracts screened for cytotoxic and antimicrobial activity on VERO cells and on *S. aureus* and *E. coli* were found to inhibit these microorganisms with a toxicity activity of 0.18 mg cm^3^ [118]. SC CO_2_ extracts of *Juniperus phoenicea* were tested on exponentially growing human CD4^+^ lymphocytes (MT-4), baby hamster kidney (BHK-21), Madin Darby bos kidney (MDBK) and human cell lines derived from liquid and solid tumor. The results showed that the extracts obtained at 200 and 300 bar were cytotoxic against different cell lines and were active against a single-stranded RNA^+^ virus [119]. SFE of volatile components of marjoram (*Origanum majorana*) and oregano showed a significantly high inhibition effects against *E. coli*, *B. cereus*, *Listeria monocytogenes*, *Salmonella typhimurium* and *Pseudomonas fluorescens.* It was confirmed that SFE with the best antimicrobial activity was correlated to the presence of carvacrol (**91**, Figure 21) [120,121,122]. Rosemary (*Rosmarinus officinalis*) volatiles are well known for their antimicrobial properties [123,124,125,126,127,128]. Antimicrobial activity of volatile fractions obtained by SC CO_2_ extraction was thoroughly studied in rosemary. The main components of this plant are α-pinene (**52**, Figure 11), 1,8-cineole (**74**, Figure 17), camphor (**81**, Figure 18) and borneol. The antimicrobial activity of rosemary SFE extract has been demonstrated against several Gram-positive bacteria (*S. aureus*, *B. subtilis*) and Gram-negative bacteria (*E. coli*, *P. aeruginosa*) [126,129]. Leaf volatiles of *Anemopsis californica* extracted by SFE and their antimicrobial activity were tested on *Streptococcus pneumoniae*, *S. aureus*, *Enterobacter aerogenes*, *E. clocae*, *Shigella flexneri*, *Klebsiella pneumoniae*, *S. typhimurium*, *Chromobacterium violaceum* and *Neissera subflava*. Some of the volatile bioactivity could be accounted for by the α-pinene in the extract [130]. 

**Figure 24:**
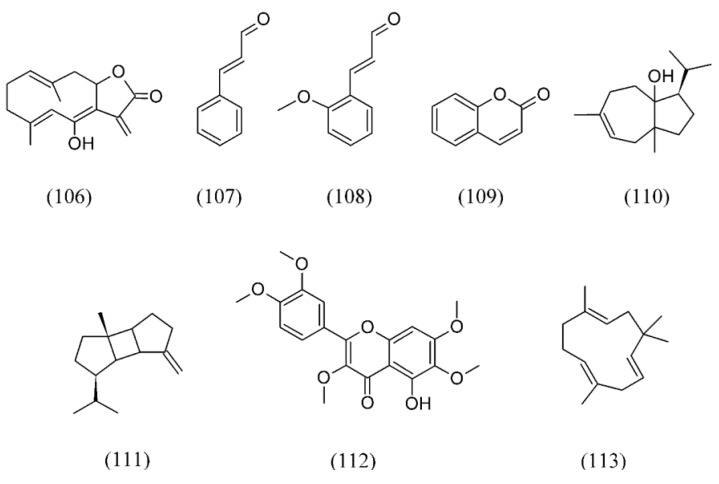
Main compounds from SFE exerting antibacterial activity.

A sesquiterpene lactone of the germacrolide type (6-*epi*-desacetyllaurenobiolide, **106**, Figure 24) extracted from laurel (*Laurus nobilis*) leaves by using SC CO_2_ was suggested to be one of the major responsible for antimicrobial activity against *S. aureus*, *B. subtilis*, *P. aeruginosa* and *E. coli* [131]. 

Various parts (barks, buds and leaves) of *Cinnamomum cassia* were extracted with SFE to evaluate antibacterial activities. SFE extracts of buds displayed significant antibacterial activity against the drug resistance pathogens, with MIC range between 0.3 and 0.7 mg mL^−1^. (*E*)-cinnamaldehyde (**107**), *O*-methoxycinnamaldehyde (**108**), coumarin (**109**) and 1,8-cineole (**74**, Figure 17) were the major antimicrobial components in the SFE extracts (Figure 24). Thus, SFE extracts of buds showed the potential value as an antibiotic substitute based on the *in vitro* antimicrobial assay [132].

Isolation of carrot fruit (*Daucus carrota* cultivar Chanteney) volatile oil by SC CO_2_ proved to be effective for its antimicrobial properties against *S. aureus*, *Enterococcus faecalis*, *B. subtilis*, *B. cereus*, *L. monocytogenes*, *Rhodococcus equi*, *E. coli*, *Salmonella enteritidis* and *P. aeruginosa*. The main component of the SF extract was carotol (**110**, Figure 24) and was most effective against Gram-positive bacteria [133].

SFE of *Curcuma aeruginosa*, *Citrus hystrix*, and *Azadirachta indica* were screened for antimicrobial activity against bacteria known to cause various types of skin infections. The antimicrobial activity was tested on *B. cereus*, *B. subtilis*, *Staphylococcus epidermidis*, *S. aureus*, *E. coli*, *Propionibacterum acnes*, and *Malassezia furfur*. The antimicrobial activity profile showed that *B. subtilis* was the most susceptible bacterial strain. According to the antimicrobial profile, SFE extracts of *C. aeruginosa* presented no significant difference in inhibitory activity on all Gram-positive bacterial strains. *A. indica* leaf extracts showed the highest antibacterial activity on *P. acnes* and *S. aureus*, a moderate activity on *B. cereus*, *B. subtilis*, and *S. epidermidis*. The SFE extracts of *C. hystrix* (stem and bark) presented the highest antibacterial activity on *B. subtilis*, moderate activity on *B. cereus* and *S. epidermidis*, and weak activity against *S. aureus* and *P. acnes*. It was concluded that the SFE extracts of *C. aeruginosa*, *C. hystrix*, and *A. indica* have the possibility to be applied as a constituent of cosmetic products and medicines, because they exhibit antimicrobial activities [134].

SC CO_2_ extracts of *Ramulus cinnamomi* were examined for their antibacterial activity against *Acinetobacter baumannii*, *P. aeruginosa* and *S. aureus* isolates by the disk diffusion method. The best extraction conditions for antibacterial activity were found to be high pressure and low temperature. Furthermore, the crude extract of *R. cinnamomi* from SC extraction showed better antibacterial activity than that obtained by ethanol extraction. The antimicrobial constituent was identified to be cinnamaldehyde (**107**, Figure 24) [135].

The volatile components and *in vitro* antimicrobial activities of Emblica (*Phyllanthus emblica*) obtained by SFE show a broad spectrum of antimicrobial activity against *S. aureus*, *B. subtilis* and *B. cereus*. β-bourbonene (**111**, Figure 24), thymol (**73**, Figure 16), and β-caryophyllene (**82**, Figure 18) were among the major compounds from the SFE extract [136,137].

The antibacterial activity of SC CO_2_ extracts from *Cordia verbenacea* (Borraginaceae), a traditional medicinal plant that grows widely along the southeastern coast of Brazil, was tested against *S. aureus*, *B. cereus*, *E. coli* and *P. aeruginosa*. The inhibitory activity of the extracts in Gram-positive bacteria was significantly higher than in Gram-negative. The most important components identified in the SF extract were artemetin (**112**, Figure 24), β-sitosterol (**55**, Figure 12), α-humulene (**113**, Figure 24) and β-caryophyllene (**82**, Figure 18) [138].

Microbial susceptibility tests revealed the great potential of *Satureja montana* volatile SFE for the growth control and inactivation of *B. cereus*, *B. subtilis*, *E. faecalis*, *E. coli*, *L. monocytogenes*, *P. aeruginosa*, *S. enteritidis* and *S. aureus*. The strongest antibacterial activity was found against *B. cereus* and *B. subtilis*. Carvacrol (**91**, Figure 21), *p*-cymene (**63**, Figure 14), thymol (**73**, Figure 16), and γ-terpinene (**53**, Figure 11) were the major compounds detected in SFE volatile extracts [139].

Garlic extracts were obtained using SC CO_2_ allowed isolation of substances (allicin, ajoene, diallyl disulfide, and diallyl trisulfide) to be tested as potential biocides against *B. cereus*, *P. aurantiaca* and *E. coli*. The results indicate that SFE sulfur-containing garlic components can be used as potential antimicrobial agent [140].

Sideritis scardica SC CO_2_ extracts were tested against *Streptococcus pyogenes*, *Streptococcus ca*nis, *Moraxella catarrhalis*, *S. aureus*, *Corynebacterium pseudotuberculosis*, *E. faecalis*, *E. coli*, *P. aeruginosa*, *K. pneumoniae*, *Pasteurella multocida* and *Haemophilus* sp. A strong to a moderate antibacterial activity of the investigated *S. scardica* SF extracts was found for all tested microorganisms [141]. 

Table 3 lists bacterial species and plant species whose SFE extract have been demonstrated antibacterial activity.

**Table 3:** List of plant species whose SFE extract exert antibacterial activity.

Bacterial species	Plant species	Reference
*Acinetobacter baumannii*	*Ramulus cinnamomi*	[135]
*Bacillus cereus*	*Allium sativum*	[140]
	*Azadirachta indica*	[134]
	*Citrus hystrix*	[134]
	*Cordia verbenacea*	[138]
	*Daucus carrota*	[133]
	*Origanum majorana*	[120,121,122]
	*Phyllanthus emblica*	[136,137]
	*Satureja montana*	[139]
	*White grape seeds*	[117]
*Bacillus subtilis*	*Azadirachta indica*	[134]
	*Citrus hystrix*	[134]
	*Daucus carrota*	[133]
	*Laurus nobilis*	[131]
	*Phyllanthus emblica*	[136,137]
	*Rosmarinus officinalis*	[126,129]
	*Satureja montana*	[139]
*Chromobacterium violaceum*	*Anemopsis californica*	[130]
*Citrobacter freundii*	*White grape seeds*	[117]
*Corynebacterium pseudotuberculosis*	*Sideritis scardica*	[141]
*Enterobacter aerogenes*	*Anemopsis californica*	[130]
*Enterobacter cloacae*	*Anemopsis californica*	[130]
	*White grape seeds*	[117]
*Enterococcus faecalis*	*Daucus carrota*	[133]
	*Satureja montana*	[139]
	*Sideritis scardica*	[141]
*Escherichia coli*	*Allium sativum*	[140]
	*Cordia verbenacea*	[138]
	*Daucus carrota*	[133]
	*Laurus nobilis*	[131]
	*Origanum majorana*	[120,121,122]
	*Rosmarinus officinalis*	[126,129]
	*Santolina insularis*	[118]
	*Satureja montana*	[139]
	*Sideritis scardica*	[141]
	*White grape seeds*	[117]
*Haemophilus sp.*	*Sideritis scardica*	[141]
*Klebsiella pneumoniae*	*Anemopsis californica*	[130]
	*Sideritis scardica*	[141]
*Listeria monocytogenes*	*Daucus carrota*	[133]
	*Origanum majorana*	[120,121,122]
	*Satureja montana*	[139]
*Moraxella catarrhalis*	*Sideritis scardica*	[141]
*Neissera subflava*	*Anemopsis californica*	[130]
*Pasteurella multocida*	*Sideritis scardica*	[141]
*Pseudomonas acnes*	*Azadirachta indica*	[134]
	*Citrus hystrix*	[134]
*Pseudomonas aeruginosa*	*Cordia verbenacea*	[138]
	*Daucus carrota*	[133]
	*Laurus nobilis*	[131]
	*Ramulus cinnamomi*	[135]
	*Rosmarinus officinalis*	[126,129]
	*Satureja montana*	[139]
	*Sideritis scardica*	[141]
*Pseudomonas aurantiaca*	*Allium sativum*	[140]
*Pseudomonas fluorescens*	*Origanum majorana*	[120,121,122]
*Rhodococcus equi*	*Daucus carrota*	[133]
*Salmonella enteritidis*	*Daucus carrota*	[133]
	*Satureja montana*	[139]
*Salmonella typhimurium*	*Anemopsis californica*	[130]
	*Origanum majorana*	[120,121,122]
*Shigella flexneri*	*Anemopsis californica*	[130]
*Staphylococcus aureus*	*Anemopsis californica*	[130]
	*Azadirachta indica*	[134]
	*Citrus hystrix*	[134]
	*Cordia verbenacea*	[138]
	*Daucus carrota*	[133]
	*Laurus nobilis*	[131]
	*Phyllanthus emblica*	[136,137]
	*Ramulus cinnamomi*	[135]
	*Rosmarinus officinalis*	[126,129]
	*Santolina insularis*	[118]
	*Satureja montana*	[139]
	*Sideritis scardica*	[141]
	*White grape seeds*	[117]
*Staphylococcus coagulans niger*	*White grape seeds*	[117]
*Staphylococcus epidermidis*	*Azadirachta indica*	[134]
*Streptococcus canis*	*Sideritis scardica*	[141]
*Streptococcus pneumoniae*	*Anemopsis californica*	[130]
*Streptococcus pyogenes*	*Sideritis scardica*	[141]

### 4.4. Antioxidant Activity

The antioxidant activity of volatile oils is of great interest because they may preserve foods from the toxic effects of oxidants. Moreover, volatile oils being also able of scavenging free radicals may play an important role in some disease prevention such as brain dysfunction, cancer, heart disease and immune system decline. Increasing evidence has suggested that these diseases may result from cellular damage caused by free radicals ([142] and references cited therein).

The antioxidant activities of the volatile and the nonvolatile fractions from *Satureja montana* obtained by SFE and by conventional techniques, hydrodistillation (HD) and soxhlet extraction (SE), were compared. SFE showed significant advantages over conventional techniques by avoiding thermal degradation and hydrolysis reactions. Furthermore, the SFE volatile oil was 15 times richer in thymoquinone (**92** Figure 21) than HD. This compound is of great importance due to its antioxidant, neuroprotective, and anti-cancer activities. The combination of carvacrol (**91** Figure 21) + thymol (**73** Figure 16) + thymoquinone (**92**, Figure 21) in SFE volatile oil may be responsible for the increase in the antioxidant activity when compared to HD, which demonstrates that, in this case, SFE was able to improve value to the final product [143]. 

SC CO_2_ extracts of ground black pepper (*Piper nigrum*) have superior reducing lipid oxidation of cooked ground pork compared to conventional extracts as measured by TEARS and hexanal concentrations. Oleoresin extracted by SC CO_2_ at 28 MPa (60 °C) was most effective in reducing hexanal concentration for up to 2 days [144]. 

Chemical compositions and antioxidant activities of essential oils from nine different species of Turkish plants, namely *Melissa officinalis*, *Rosmarinus officinalis*, *Cuminum cyminum*, *Piper nigrum*, *Lavandula stoechas* spp., *Foeniculum vulgare*, *Pimpinella anisum*, *Thymus serpyllum* and *Liquidamber orientalis*, have been studied using volatile oils obtained by SC CO_2_ extraction. In the DPPH assay, *R. officinalis*, *C. cyminum*, *P. anisum*, *T. serpyllum* and *L. orientalis* volatile oils obtained by SC CO_2_ extraction showed higher antioxidant activity than steam distilled extracts [145].

## 5. Process Considerations

We now turn to the process modeling of SFE. In front of the relative technical simplicity of traditional extraction techniques of volatile oils from aromatic plants as hydrodistillation and steam distillation, the SFE can be described as a five-step process: (1) penetration of matrix; (2) SCF solubilizes the solutes inside the pores; (3) intraparticle (or internal) diffusion of the solutes takes place until the external surface; (4) external (or film) diffusion of the solutes from solid-fluid interface to the SCF bulk; and (5) precipitation of target solutes in the trapping system by changing the pressure and/or temperature of the effluentdd. Each part of the process has to be carefully optimized in order to obtain a desired quality and yield [10,11].

**Figure 25:**
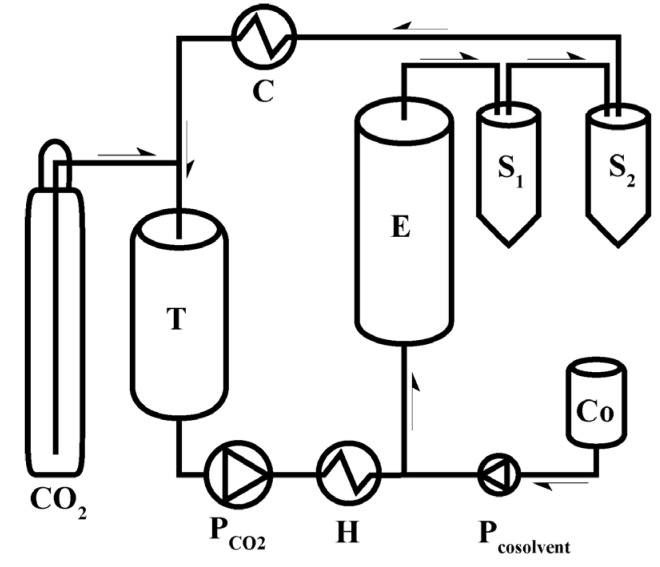
Diagram of a supercritical fluid extraction pilot plant equipped with two fractionation cells. (T) Storage Tank; (PCO_2_) CO_2_ Pump; (H) Heat exchanger; (Co) Cosolvent tank; (Pcosolvent) Cosolvent Pump; (E) Extraction vessel; (S1-S2) Separation cells; (C) Condenser.


The extraction scheme is illustrated in Figure 25. First, the liquid carbon dioxide is pumped through a heat exchanger to reach the system at supercritical state, after the SC CO_2_ is uniformly pumped in the extractor where the dry and ground plant material forms a fixed bed of solid matrix. The extraction can be performed in static (with no follow-through) or dynamic (with follow-through) mode or in a mixed approach. During extraction, the supercritical solvent passes through the plant matrix bed and dissolves the soluble compounds. The mixture solvent-plant solutes is separated in flash tanks (cyclonic and gravimetric separators) usually changing drastically the solvent power of CO_2_ by depressurization or temperature change or both. Then, CO_2_ is cooled at liquid state and compressed to return to the extractor [11,146].

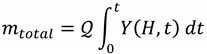
(1)

The kinetics of SFE extraction can be described through the Overall Extraction Curve (OEC) equation. The formula [Equation (1)] describes the relation between the extracted solute (mtotal) with the time of extraction and amount of flowed solvent, where Q is the flow rate of the supercritical fluid, Y(H, t) is the ratio of solute mass to solvent mass at a given time (t) at the extractor outlet (H). 

Typically, the OEC can be divided in three distinct parts: the first of constant-extraction rate period (CER), the region of curve with a linear trend; the second of falling-extraction rate period (FER), and the third of diffusion controlled rate period (DCR). Generally, 50–90% total solute is extracted during the CER, therefore the estimation of costs of manufacturing must take in account the best operational conditions allowing the highest solute extraction in the shortest time or lowest amount of required solvent [11,127,147,148,149].

However, an additional level of difficulty is the thermodynamic description of the extraction process. Complex mathematical modeling is required because in presence of a permeable material as the plant material, the supercritical fluid penetrates the matrix (by viscous flow-hydrodynamic process based on P difference) when operation starts. After that the SCF stays inside the solid, and the transport mechanism of the solutes in steady state is “unimolecular diffusion”. The presence of solutes in the supercritical solvent modifies the system. Critical pressure and temperature become a function of system composition even for the presence of co-solvents. The thermodynamic description has to follow the dynamic mass transfer of solutes into the solvent. In real systems, due to the complex chemical composition of aromatic plants, the corresponding thermodynamic diagrams is of unapproachable complexity, far from practical extraction planning [150,151]. The two component phase diagrams can describe adequately the system because the aim of the process is separate two pure phase, the solvent and the solute mixture [152,153,154].

The first step in the setup of an SFE requires knowledge of the mass-transfer mechanisms, kinetics parameters and thermodynamic restrictions related to plant materials with complex cellular structure [10,146]. Several mathematical models have been developed in the last 15 years. In 1997, Reverchon proposed the classification of models in: (1) empirical models, (2) models based on heat transfer analogy and (3) models based on integration of differential mass balance equations [1].

The first group is characterized by the simplicity of the formulae; it considers the requirements of extraction yield on the extraction time without considering information about the different types of matrices. The parameters have no physical meaning therefore this model is not applicable to different conditions or for scale-up [29,155].

The second group of models is based on heat and transfer analogy. The model compares the matrix to spherical particles in a uniform medium and the mass transfer to the cooling of the spheres. The equations describe the material concentration across an internal surface [1,155]. These models give good yield approximation, even if different particle size are considered; but if particle charge distribution is considered, the model tends to overestimate extraction yield because it does not keep in account the interaction between particles [1].

The third group of models is based on the integration of differential mass balance equation. This model group has the strongest physical validity, including mass transfer coefficients of fluid and solid phases. Moreover, this group takes into account also the characteristics of the plant matrix [155]. Parameters like phase equilibrium, mass transfer resistances and flow patterns allow description of time-dependent concentration profiles for both solid and fluid phases [1,29]. Thus the second and third group can be included into one group of phenomenological models [29]. 

Several authors proposed the grouping of models based on different internal mass transfer mechanisms, depending from the target compound class and the matrix characteristics. The considered mechanisms are classified in: diffusion models, desorption models, shrinking core models and BIC (broken and intact cells) models [1,29,34]. 

Finally, in case of aromatic plants, the different glandular structures that accumulates the target compounds and the relevance to adopt microstructure-based models in order to accurately describe flavors and fragrances SFE from different categories of aromatic plants have been considered [29].

The versatility of SC CO_2_ as extraction technology is linked to the possible drastic change of solvent power through the simple change of pressure and temperature. The range of variation of SC CO_2_ density is relatively wide, from 0.2 g cm^−3^ at 8 MPa and 60 °C to 1.0 g cm^−3^ at 50 MPa and 40 °C [6]. Furthermore, the increase of temperature leads to reduction of density of supercritical fluids but, on the other hand, the increase of temperature affects the volatility of target compounds. For volatile oil extraction through SC CO_2_, small changes in temperature can cause significant changes in solubility with a non-linear relationship [156]. Whereas the operative pressure is the main parameter that influences the fluid density and therefore the solvent power of supercritical fluid, the effect of temperature depends on the nature of plant material and has to be determined case by case [157].

For the analysis of solubility of target compounds and for the design of extraction process, four parameters are extremely helpful in the understanding of solute behavior in supercritical fluids. The miscibility or threshold pressure, that is the pressure at which the solute starts to be transferred into the supercritical fluid; the pressure of maximum solubility of solute; the fractionation pressure range, that is the pressure region between the miscibility and maximum solubility pressures and; the physical properties of the solute, particularly its melting point. The determination of the last two parameters allows to define the best conditions for solubility and selectivity, because compounds diffuse better above their melting points and an operative pressure between miscibility and maximum solubility increases the selectivity of extraction [10,158].

For the analysis of pressure and temperature effects on extraction, the global yield isotherm has been widely used. Global yield can be referred to a single target compound or to the global mixture of compounds. This parameter is closely related to the solubility of the solute in the supercritical fluid [11,159,160]. Moreover, the solubility of target compounds can be determined also from the slope of the linear portion of the extraction curve in the stage of constant-extraction rate period (CER) [146].

Beyond the extraction parameters related to the engineering aspects such as pressure, temperature and flow rate, other factors related to the nature of plant material can influence the SFE. The particle size, shape, surface area, porosity, and moisture level of extractable solutes are variables that depend on the nature of the matrix or pretreatment of the plant material. As a rule, the smaller is the particle size of plant material the higher it will be the exposed surface for SC CO_2_ penetration and solute heat transfer. However, the excessive grinding of the material might produce an extraction bed extremely thick and the SC CO_2_ could find fast tracks inside the extractor (fluid channeling effect), thus reducing the contact with the plant material [10,11].

Moreover, the moisture content of the solid material influences not only the extraction quality and yield but also the fluid dynamics of the solvent. Water can act as co-solvent by interacting with the supercritical solvent and by changing the overall polarity of the fluid. However, extracted water can increase the formation of ice blockages. Therefore, drying the raw material it is recommended in order to have a water content of around 4–14%. 

Co-solvents can act through two hypothetical mechanisms: solute–co-solvent interaction, and matrix swelling which facilitates the contact of the solutes with the solvent. The co-solvents do not have absolute mechanism of action; their effects are related to the type of co-solvent, plant material and target compounds. Studies about the effects of co-solvents at constant pressure and temperature evaluated the extraction efficiency of different modifiers at increasing percentages for volatile oil extractions. The addition of methanol, ethanol or halogenated co-solvents reduces the number of extracted terpenes with respect to pure SC CO_2_ for *Perovskia atriplicifolia* but increases the extraction selectivity [157]. However, the use of co-solvents, especially at high percentages, leads to a biphasic system that will change the critical parameters of the mixture. The most common co-solvent are short chain alcohol among which ethanol and methanol predominate. Usually, they are added in a percentage that varies from 1% to 15% [89,161,162,163]. However, safer and less harmful solvents that are easy to remove, or recover, are gaining popularity in agronomic applications of SFE [146] and only ethanol and water have the lower toxicity in the final extract. 

Effective models of extraction and experimental tests are crucial key points to determine the basic mass transfer data necessary for scale-up procedures. The relative slow diffusion at industrial level of SFE is due to the difficulty to setup extraction conditions and to the haziness of scale up from laboratory scale to industrial scale. 

The feasibility study of SFE requires the evaluation of several objectives:

1. The solubility and mass transfer of target compounds in the SC CO_2_. The practical analyses shall verify if SFE is the suitable technique for the extraction of the target compounds [11];
2. Evaluation of extract quality. Pressure and temperature can seriously influence the composition of the final extracts;
3. Pressure drop effect;
4. Process optimization to obtain the best ratio between yield and quantity of solvent amount and time of extraction;
5. Scale up optimization

This information is used to estimate the cost of manufacturing (COM) [13,164,165].

The design of industrial-scale equipment is usually preceded by laboratory (less than 2 L extractor vessel) and pilot-scale systems (2–100 L extractor vessel). Often the pilot-scale system is skipped, and work goes straight from the laboratory to industrial production. Usually, the laboratory scale experiments are performed in grams scale or smaller, often with instruments that have a design which is far from the industrial plan [13,93]. However, with sound lab data and assessment of pilot scale factors the design of industrial sized equipment is much more efficient. In fact, the scale up requires the adjustment of system geometries, fluid dynamics parameters and other factors that can influence extraction. [146,166]. One of the most delicate questions detected with pilot plant experiments is the existence of channeling. For instance, the decrease in the extraction of lycopene yields with an increase in flow rate of SC CO_2_ can be attributed to channelling effects that inhibits CO_2_ dissusion in to the sample. When the SC CO_2_ flow rate is increased, it flows through the sample at high velocities and instead of diffusing through the sample matrix, it flows around the sample through channels, thus limiting the contact necessary for extraction of the desired compound [167].

Another important issue is the production of sufficient quantities of extract in order to test its quality, reproducibility of composition, biocompatibility (if applicable). Many times samples must be characterized and evaluated, mainly in food, pharmaceutical and cosmetic industries, and this cannot be achieved with quantities obtained at lab scale. For instance, in cosmetic applications, one needs quantities in the order of kilograms.

## 6. Conclusions

SFE is a technology that allows extraction of a wide range of diverse compounds from a variety of plant matrices. SC CO_2_ is suitable for the extraction on many non-polar to moderately polar compounds, while more polar compounds can be extracted with subcritical water or by the use of SC CO_2_ and co-solvents. SFE can be considered a sound cleantech strategy to extract natural compounds with an undisputed environmental friendliness. This is due to the non-toxic nature of fluids used such as CO_2_. SFE studies start from laboratory scale and scale-up developments lead to industrial plants of huge dimension and yield. There are numerous advantages over classical liquid solvent extractions including rapidity, selectivity, cleanliness, low solvent volumes required and possibility of manipulating the composition of the extract through selective precipitation of classes of compounds. However, this technology is costly and requires a careful business plan contemplating the cost/effective analysis of the molecules and phytocomplexes to be extracted.

Despite the evident advantages and applications of SFE technology, there still is room for improvement in the application arena. Information on the influence of operating parameters (such as the change in temperature and pressure) on particle size and its morphology still require thorough studies. Since SFE has added a new dimension to the pharmaceutical and nutraceutical research and formulation development its potential must be exploited technologically and economically to provide new sustainable and reliable natural resources contributing to human and environmental quality of life.
